# Plasmonics of 2D Nanomaterials: Properties and Applications

**DOI:** 10.1002/advs.201600430

**Published:** 2017-02-16

**Authors:** Yu Li, Ziwei Li, Cheng Chi, Hangyong Shan, Liheng Zheng, Zheyu Fang

**Affiliations:** ^1^ School of Physics State Key Lab for Mesoscopic Physics Peking University Beijing 100871 China; ^2^ Academy for Advanced Interdisciplinary Studies Peking University Beijing 100871 China; ^3^ Collaborative Innovation Center of Quantum Matter Peking University Beijing 100871 China

**Keywords:** 2D materials, light‐matter interactions, optoelectronics, surface plasmons

## Abstract

Plasmonics has developed for decades in the field of condensed matter physics and optics. Based on the classical Maxwell theory, collective excitations exhibit profound light‐matter interaction properties beyond classical physics in lots of material systems. With the development of nanofabrication and characterization technology, ultra‐thin two‐dimensional (2D) nanomaterials attract tremendous interest and show exceptional plasmonic properties. Here, we elaborate the advanced optical properties of 2D materials especially graphene and monolayer molybdenum disulfide (MoS_2_), review the plasmonic properties of graphene, and discuss the coupling effect in hybrid 2D nanomaterials. Then, the plasmonic tuning methods of 2D nanomaterials are presented from theoretical models to experimental investigations. Furthermore, we reveal the potential applications in photocatalysis, photovoltaics and photodetections, based on the development of 2D nanomaterials, we make a prospect for the future theoretical physics and practical applications.

## 1 Introduction

Materials present distinct properties as their size reduce into low‐dimensional regime, where atoms and electrons are restricted to limited free degree, involving strong light‐mater interaction, ultrahigh electric conductivity and excellent mechanical flexibility. In the recent years, many kinds of 2D materials are analyzed ranging from graphene,^1^ layered transition metal dichalcogenides (LTMDCs) to hexagonal boron nitride (hBN),^2^, ^3^ their excellent optoelectronic properties make a wide range of optoelectronic applications become possible.^4^ Semi‐metallic graphene interacts with photons in a large range of energy,^5^, ^6^ considering its ultrahigh carrier mobility at room temperature (higher than 10^4^ cm^2^V^−1^s^−1^) and high degree of optical transparency (approximately 97.7%), graphene shows unique capability of supporting and tuning surface plasmons which can be utilized in light detection applications and new light‐involved electronics design.^7^, ^8^, ^9^, ^10^, ^11^, ^12^, ^13^, ^14^, ^15^ MoS_2_ and WSe_2_ are two typical 2D semiconductors, which belong to LTMDCs. Compared with graphene, the carrier mobility of few layered semiconductors is relatively low, but it is much higher than that of the bulk semiconductors as gallium arsenide (GaAs) or silicon of the same thickness.^16^, ^17^ The optic bandgaps of LTMDCs range from 1.0 to 2.5 eV, making them suitable for light emitters and absorbents in a wide spectrum.^18^ However, due to the limited light‐harvest ability and manipulating possibility, efficient utilizations of 2D material are far from practical applications.

To solve this problem, the strategy of integrating 2D materials with plasmonic nanomaterials attracts much interest. Owing to the increased light‐harvesting efficiency and enhanced near‐field intensity, hybrid 2D materials show exceptional potential for photocatalysis and photodetections.^19^, ^20^ Plenty of plasmonic structures which have been intensively studied can be utilized to actively control light‐matter interactions, and enable a wide reign of optoelectronics applications.^21^, ^22^, ^23^, ^24^ Moreover, plasmonic hot electrons, which inject into 2D materials and rapidly change the carrier intensity in the materials, enable even more applications for photovoltaic cells and photocatalysis.^25^, ^26^ Furthermore, coupling effects between excitons and plasmons in the 2D materials/plasmonic structures hybrid system are of growing interest considering the existence of different kinds of quasiparticles in the system, and inspire more profound studies for light‐matter interactions.^27^, ^28^

In this review article, we start from basic optical properties of 2D materials, focus on the properties and tuning methods of plasmons in both 2D materials and hybrid nanomaterials. By discussing properties of individual 2D materials and the coupling effects especially strong coupling effects with the plasmonic integration, we overview the strategies of making use of plasmonic properties and excitonic irradiations to achieve different photoelectric reactions and applications.

## 2 Plasmonic Properties of 2D Nanomaterials

The atomic thin 2D nanomaterial sheet presents significant light‐matter interaction phenomena, owing to quantum confinement effect, their electronic structures and optical properties are distinctive from their bulk morphology.^29^ Because of those advanced electronic and optical properties, plasmonic properties of 2D nanomaterials exhibit more attractive characteristics. Metallic graphene support SP mode in the infrared regime while excitonic LTMDCs/plasmonic structures hybrid system exhibits profound coupling effect, which induces more interesting plasmonic properties of 2D nanomaterials.

### 2.1 Electronic and optical properties of 2D nanomaterials

**Figure 1:**
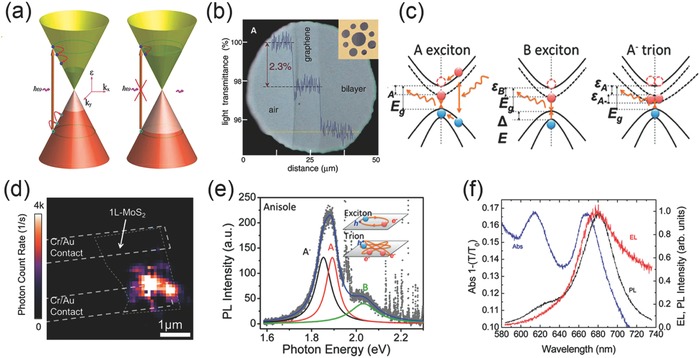
a) Schematic view of the light absorption process in graphene: excitation, relaxation and absorption block. Reproduced with permission.^[30]^ b) Optical image of graphene and its bilayer. (Inset) the experimental sample design. Reproduced with permission.^[8]^ Copyright 2008, AAAS. c) Schematics of A, B exciton and A− trion formation in MoS_2_ monolayer. Reproduced with permission.^[37]^ Copyright 2014, ACS. d) Electroluminescence mapping of monolayer MoS_2_. Reproduced with permission.^[43]^ Copyright 2013, ACS. e) PL spectrum. (Inset) the schematic view of MoS_2_ monolayer comprising trion and exciton component. Reproduced with permission.^[37]^ Copyright 2014, ACS. f) Comparing absorption (Abs), EL, and PL spectra of monolayer MoS_2_. Reproduced with permission.^[43]^ Copyright 2013, ACS.

Optical properties of 2D materials emerge from their distinctive electronic structures. For the graphene, electrons can travel on the sheet of comb‐like carbon lattice, which can be well described as linear dispersed ideal Dirac fermions near the Fermi energy.^30^ The light absorption process of graphene can be divided into two steps, as shown in **Figure** **1**a. When the graphene is illuminated by light, electrons in valence band are excited into the conduction band, then a hot Fermi‐Dirac distribution of thermalized hot electrons generates immediately (≈150fs). The new distribution of hot electrons impedes a part of original interband transitions within 1 picosecond, which decreases further light absorption. For the temperature of electron in the distribution, the light with the frequency of k_B_T_e_/ħ is mainly blocked from absorption. After that, hot electrons and holes are cooled down by the scattering of interband photons, and mainly recombine to each other until the electron‐hole distribution reach equilibrium. From this process, we can notice that the absorption of graphene is a constant related with its inner property and the absorption of multilayer graphene is *πα* (≈2.3%) for each atom layer because of the weak interlayer coupling of different 2D electron gas layers (Figure 1b).^8^ Notably, the absorption constant follows the function as:

(1) $$1\;-\;{\left(1\;+\;0.5\pi \alpha \right)}^{-2}\;\approx \;\pi \alpha \;\approx \;2.3\%$$

In which α = 2*πe* ^2^/*hc* ≈ 1/137 is the conventional fine structure constant (*h* is the Plank constant, *c* is the speed of light). Remarkably, this linear‐dispersion property and high‐efficient light trapping characters make graphene useful for transparent electronic devices, ultra‐sensitive photodetectors and other high‐performance optoelectronic devices, notably, the special optical property changes under high excitation intensity. Further research shows that when the concentration of hot carriers increase and become higher than the intrinsic carrier density in graphene. The states near the edge of the conduction and valence bands are filled because Fermions cannot occupy an exactly same state, making light absorption be saturated. Compared with traditional semiconductors like silicon and GaAs this unique saturable character of graphene under strong light excitation makes it promising in the high speed communication field as ultrathin saturable optical fiber absorber, and leads to a new generation of highly integrated low‐noise optical communication system with very low cost.^31^, ^32^

Different from metallic graphene monolayer, semiconducting LTMDCs are chemical compounds comprised of group IV, V and VI elements, which are transition metal element and chalcogen. They follow the compound form as X–M–X, which is two chalcogen layers bond with a transition metal monolayer in the middle, the corresponding atom number ratio is 2: 1 while the conformation can be various.^33^, ^34^ Those sandwich atomic structures, which we call as LTMDCs are bonded by covalent bond while different layers of LTMDCs can form the bulk by weak interlayer bond, and support unique distributions of electrons. Thus, the absorption properties of LTMDCs are distinctive from the bulk form or the comb‐like graphene.^35^

Monolayer MoS_2_ is a kind of semiconducting LTMDCs and researches show that the absorption properties are directly influenced by the electronic structure, which follows the rule of quantum confinement. Different from its bulk form, the band gap of monolayer MoS_2_ transforms from original indirect type to direct type as the atom layers decrease to one, which attributes to quantum confinement and the orbital hybridization of MoS_2_ atoms, especially p_z_ orbitals of S atoms and d orbitals of Mo atoms, while the direct excitonic transitions rarely change. Notably, d orbitals of Mo atoms play a dominant role in the Brillouin zone K‐point conduction band state. The middle Mo atoms layer restricted between the two layers of S atoms makes the interlayer coupling weak, thus direct excitonic transitions do not change with number of MoS_2_ layers. However, the states around Γ‐point are also influenced by p_z_ orbitals of S atoms which construct as two layers on both sides of MoS_2_ monolayer. It enables the hybridized state to interact with adjacent MoS_2_ layers easily, thus the band gap at Γ‐point shows significant layer dependent properties.

The electronic structures and optical properties of semiconducting MoS_2_ can be further analyzed as many body systems controlled by the Coulomb interaction of electrons and holes.^36^ Specifically, the combination of one electron and one hole forms a quasiparticle named exciton, while three particles (two electrons and one hole, or one electron and two holes) can comprise a trion, or charged exciton (Figure 1c).^37^ The behaviors of excitons and trions have strong influence on optical phenomena including absorption and light emission. Reported by many groups, spin‐orbit coupling effect induces the valence band edge of monolayer MoS_2_ to split, and the maximum of split locates at the K point of the Brillouin zone, while the minimum of conduction band also locates at K point.^38^, ^39^, ^40^ At the K point of Brillouin zone, the split transition band corresponds to two strongly bound A exciton and B exciton, of which the split band transition energy in monolayer MoS_2_ is approximately 1.92eV and 2.08eV, and induces two separated absorption peaks in red to near infrared region. The optical transition bandgap energy should take exciton binding energy in consideration, which increases under the influence of the dielectric constants, so less energy is needed to generate an exciton than the transport bandgap energy. Theoretical calculation from the Bethe‐Salpeter equation (BSE) also proves that absorption spectral reflects the movement of A, B exciton peaks when the comprising elements of LTMDCs are changed .For heavier chalcogen species, the effective exciton mass along with the dielectric screening increases, thus the exciton binding energy decreases because the transition energy equals to *μex*/ε2 approximately, which corresponds to the redshifts of the A and B exciton peaks. The changes of metal atom do not have significant influence on the transition gap of A exciton. But for B exciton, the peak position is very sensitive to the choice of metal atoms owing to the spin‐orbit coupling effects.

As mentioned above, the behaviors of excitons and trions play an important role in LTMDCs luminescence properties. Based on the methods of excitation, there are mainly two categories of 2D material luminescence, which are electroluminescence (EL) and photoluminescence (PL). For monolayer MoS_2_, which is a kind of direct bandgap semiconductor, strong EL can be observed under electric excitation. By applying voltage on the source and drain electrodes, electrons are injected into conduction band and become strongly bended at the contact area between MoS_2_ and metal on a high bias condition. This injection process is able to generate excitons effectively, further induces hot carriers which are electrons in majority to be backscattered as well as accumulated on the drain electrode. As a result, the minority carriers of holes in the monolayer MoS_2_ sheet will be raised from the drain electrode which result in the recombination of electrons and holes and emit photons. Figure 1d is the EL distribution mapping of the monolayer MoS_2_ device, as shown in the picture, EL is not homogeneously emitted from the monolayer MoS_2_ but shows a strong accumulation on the metal electrodes. Notably, the area with the most intensive EL signal is the source of carrier injection, where the efficiency of exciton generation reaches its maximum, because the strong band deformation plays dominant role in the hot carrier excitation.

Another category of luminescence is PL which comes from the radiative recombination of hot electron‐hole pairs excited by photons.^41^, ^42^, ^43^ As shown in Figure 1e, a typical PL spectrum consists of two noticeable peaks, which is actually believed to originate from the radiative recombination of the A‐ trion (∼1.85 eV), A exciton (∼1.90 eV), and B exciton (∼2.03 eV), while peaks of A‐ trion and A exciton are indistinguishable at room temperature. The absorption, high‐bias induced EL and PL spectral of a monolayer MoS_2_ sample are well connected (Figure 1f). In the absorption spectrum, there are two main peaks at wavelength of 610 nm and 670 nm, while in the PL spectrum, there are two corresponding peaks at 620 nm and 680 nm of the same origin. The Stokes shift of peak position is due to the inhomogeneity of dielectric environment, which influences the surface interaction of monolayer MoS_2_ and further changes the binding energy of exciton via electron‐hole Coulomb interaction. Notably, the emission peak of B exciton at the wavelength of 680nm is identical in both EL and PL spectral, which indicates that the exactly same state is excited in the material. While the difference between EL and PL mechanism still exists because the electrical power density is insufficient to excite the B excitonic state, which is at a higher energy state, thus the peak at 620nm in PL spectrum does not exist in EL spectrum.

For LTMDCs as monolayer MoS_2_ and MoSe_2_, which belongs to direct‐bandgap semiconductors, by either electric or optic method, hot electrons and holes are excited, then they recombined and emit photons.^44^, ^45^ Since this process is much more efficient than that in the indirect‐bandgap semiconductors, LTMDCs show significant potential to be utilized as active light‐emitting layer in future highly‐integrated and flexible optoelectronic devices. For EL properties of monolayer MoS_2_, the emission of light comes from electric excitation with high quantum efficiency, which makes it promising for ultrathin and economical optoelectronic devices as light‐emitting diodes (LEDs) and lasers. While in PL phenomenon, LTMDCs absorb light and generate electrons as well as holes, then emit light when they recombine, which also shows unique potential as a component in new generation of optoelectronic devices.

As for metallic graphene, which is believed to be a zero band gap 2D material, light emission phenomena are also been found and studied by introducing a band gap, or by strong excitation of ultra‐fast laser to the material. The gapless and linear dispersed properties of graphene show unique negative dynamic conductivity in terahertz (THz) spectral region making it possible to be a terahertz laser emitter.^46^, ^47^ Researchers used an ultra‐fast pulsed fiber laser at the wavelength of 1550nm to illuminate heteroepitaxial graphene on Si substrate and experimentally observed the ultra‐fast relaxation and studied corresponding recombination dynamics of hot carriers induced by the laser beam and further studied the light emission properties and the hot carrier mechanics of exfoliated graphene under the excitation of a pulse laser. Using a time‐domain spectroscopic study, which includes an optical pump and terahertz/optical probe technique, they proved that the incidental optical field will be strongly amplified by the graphene sheet. Furthermore, properties of graphene emission strongly depend on the intensity of pumping power, which further influence the non‐equilibrium carrier relaxation recombination process in the material. Combining with the III‐V semiconductors and metal nanostructures, graphene is a promising candidate for the new generation of terahertz (THz) electronics as flexible, compact, tunable and coherent light emitters and detectors for THz wave.

### 2.2 Surface Plasmon of 2D Nanomaterials

Plasmons are a kind of quasiparticle, which describe the collective oscillations of the electron gas in many metals and semiconductors. Metallic plasmons have been profoundly studied in the past decades, since the marvelous electromagnetic and optic properties they show.^48^, ^49^, ^50^, ^51^ It is proved that the boundary conditions between metal and dielectric decide the interaction between polarizing electromagnetic field and the collective oscillation of free electrons at the surface of metal. Under some conditions, the collective oscillation becomes in phase with the outer optic field at certain frequencies. As a result, the incident light waves are trapped on the interface, which we call them as surface plasmon polaritons. Since the energy is mostly localized on the surface of metal, strong electromagnetic waves are further induced at the near filed, additionally, we divide the plasmon effect into localized surface plasmons (LSPs) and surface plasmon polaritons (SPPs) according to their propagation condition. Until now, quantities of plasmonic nanoparticles and nanostructures have been deeply studied, for example, nanorods, nanocavities, rings and many other patterns lead to various light‐matter interaction. Plasmonic focusing structures as in‐plane Fresnel zone plates (FZPs), circular nanocorral structures and spiral structures with distinctive spin‐state selective character. Plasmonic Fano resonance structures as sliced Ag nanodisks, dolmen‐shaped structures as well as chiral Fano resonance structures which enable even stronger super chiral field. Plasmonic waveguide coupling structures as nano‐antennas and dielectric nanoribbons which support the hybridization of plasmonic coupling. As the progress of fabrication technique and theoretical methods of surface plasmons control, extensive applications as perfect light absorbent, emitters, optical sensors and many other utilities are discovered. Along with the electromagnetic field enhancing nature, NPs and nanostructures give us perfect inspirations to manipulate light‐matter interaction in the nanoscale region.

Graphene plasmons, however, are quite different from those in metal structures.^52^, ^53^, ^54^ As mentioned above, this zero band gap semimetal supports significant light‐matter interaction of high efficiency, and shows undispersed absorb properties.^55^, ^56^, ^57^ Furthermore, the plasmonic condition can be modified by electrical and chemical doping or hybridizing graphene with other 2D materials or traditional plasmonic nanostructures.^58^, ^59^ Among several kinds of intrinsic plasmon modes in graphene, there is longitudinal mode which is also defined as transverse magnetic mode and the polarizing electric field is parallel to the wave vector *q*. Transverse collective mode, which is also called as transverse electric mode is of higher energy than the Pauli‐blocking level for interband absorption. The plasmonic frequency of transverse electric mode in monolayer graphene is between 1.667*E_F_*/*ħ* and 2*E_F_*/*ħ*, according to this, the plasmonic mode frequency can be freely modified over a broad range, by applying gate voltage and changing the charge carrier density. While the energy of the longitudinal plasmon mode is nearly zero at a very long wavelength, which makes it a gapless mode.

Since electrons in graphene sheet can be regarded as two dimensional Dirac Fermions, to describe this collective oscillating system of Dirac Fermions, the Hamiltonian at a low energy can be shown as follows:

(2) $$\hat{H}\;=\;\nu_{F}\sum_{i}\;\sigma •\;p_{i}\;+\;\frac{1}{2}\sum_{i\;\neq \;j}\;\frac{e^{2}}{\varepsilon \left|r_{i}\;-\;r_{j}\right|}$$

**Figure 2:**
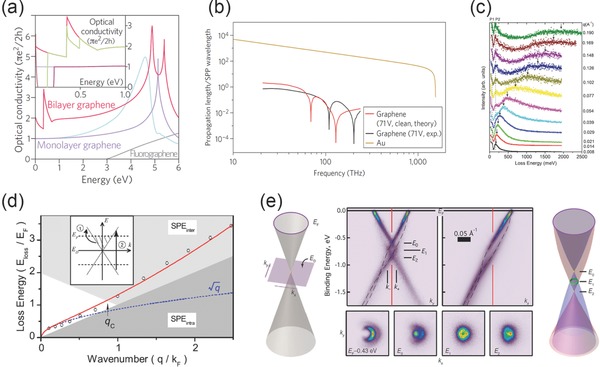
a) Optical conductivities of pristine monolayer and bilayer graphene, doped monolayer and bilayer graphene and fluorographene. (Inset) enlarged spectral in the low energy region. Reproduced with permission.^[52]^ Copyright 2012, Nature Publishing Group. b) Plasmonic propagation length and SPP wavelength of graphene and gold. Reproduced with permission.^[53]^ Copyright 2012, Nature Publishing Group. c) Surface plasmon loss spectrum of graphene in different momentum transfer condition. d) Plasmon dispersion of graphene and free 2D electron gas at the same electron density. (Inset) the band structure of graphene. c,d) Reproduced with permission.^[14]^ Copyright 2008, APS. e) Angle‐resolved photoemission spectra of doped graphene on SiC and schematic view of interacting and non‐interacting Dirac energy spectra. Reproduced with permission.^[63]^ Copyright 2010, AAAS.

In this simplified function, *v_F_* stands for the Fermi velocity, which is approximately 10^6^ m/s, **σ** is the Pauli matrix, while ***p*** follows the definition as $p_{i}\;=\;-\frac{ih}{2\pi }\nabla_{r_{i}}$, which is the canonical momentum of the specific electron numbered as *i*. The second item of this equation indicates the interaction between every other electron in this system and the single electron numbered as *i*. More specifically, this electron‐electron interaction in the two dimensional system can be quantified as potential *u*(|**r** *_i_* − **r** *_j_*|), which is decided by the length of the distance between each two electrons numbered as *i* and *j*. Since the interaction condition is very sensitive to the environmental dielectric constant, suppose a graphene monolayer which is exposed to two kinds of dielectric media, the interaction potential becomes $u(r_{ij})\;=\;\frac{2e^{2}}{(\varepsilon_{1}\;+\;\varepsilon_{2})r_{ij}}$. For doped graphene, the distance between two electrons is represented as ***r*** *_i_* – ***r*** *_j_* ≈1/*k_F_*, where *k_F_* stands for the Fermi wave number. Thus, the second term of the function follows the form as *e* ^2^ *k*_F_/ε, considering the first term of the function which describes the kinetic energy in the form of *hv*_F_ *k*_F_/2π, the ratio connecting this two items in the function should follow the definition as *cα*/*εv_F_*, which links with the fine structure constant. Notably, α is the conventional fine structure constant as discussed above, which indicates the link between absorb properties and intrinsic plasmons of graphene. Furthermore, by changing the dielectric constant of environmental media ε, electromagnetic properties of graphene can be well modulated, which makes graphene promising for more optoelectronic devices. To further discuss the plasmonic properties of graphene under illumination, we consider perpendicular incident light with the energy flux of $\frac{c}{4\pi }|\Theta {|}^{2}$, the initial and final states are defined as |*i*> and |*j*>, the absorbed energy of unit area graphene follows as 2*πω*|*M*|^2^ *D*, where *M* is the element of matrix reflecting the reaction between light and electrons, *D* is the density of states and it is linear with ε. The Hamiltonian which describes the interaction between incident light and electrons is in the term of $v_{F}\;\sigma \cdot \frac{e}{i\omega }\;\Theta$. By analyzing initial and final states, the interacting matrix can be calculated as $\frac{1}{8}e^{2}v_{F}^{2}\frac{|\Theta {|}^{2}}{\omega^{2}}$, from which we can further conclude the absorbed energy $W_{a}\;=\;\frac{\pi e^{2}}{2h}|\Theta {|}^{2}$. As a result, the optical conductivity $G\;\;\equiv \;\;\frac{W_{a}}{|\Theta {|}^{2}}$ is theoretically calculated and equals to $\frac{\pi e^{2}}{2h}$, which is independent with the properties of graphene sheet, and the absorption can also be derived as *πα*, meeting well with the experimental results as shown in **Figure** **2**a.

Furthermore, in the long wavelength region, collective oscillations of electrons are restricted by the particle number conservation and current conservation laws, thus the corresponding dispersion relation of plasmon frequency ω and wave vector *q* in monolayer graphene can be theoretically calculated by the method of Random Phase Approximation (RPA) as:

(3) $$\omega \left(q\;\to \;0\right)\;=\;{\left(\frac{ke^{2}\nu_{F}q}{\varepsilon_{g}\hbar }\sqrt{\pi ng_{s}g_{v}}\right)}^{\;\frac{1}{2}}$$

In this function, n stands for the concentration of electrons in the conduction band of graphene, while *g_v_* presents the valley degeneracy factor and is equal to 2. The appearance of Dirac constant *ħ* in the function shows the non‐classical plasmon dispersion property of monolayer graphene in the long wavelength region. Thus, compared with the classical plasmonic oscillation model, plasmons in monolayer graphene layer show unique dispersion characters, which can be well explained by a massless Dirac plasma model differs from any classical analogy. Apart from un‐patterned graphene monolayer, plasmonic properties of graphene nanoribbons are also researched profoundly in the recent years, for the zigzag type of graphene nanoribbon, frequency spectrum of plasmons can be calculated numerically. While for the type of armchair nanoribbon, the method of one‐band approximation within the RPA at γ = 0 can be utilized under the condition of Re[ε(*q_x_, ω*)] = 0, and the corresponding plasmon frequency ω follows the function as:

(4) $$\omega^{2}\;=\;\nu_{F}{}^{2}q_{x}{}^{2}\;-\;\frac{V_{0,\;0}\left(q_{x}\right)f_{1}\left(q_{x},\beta ,\mu_{g}\right)g_{s}\nu_{F}q_{x}}{\pi \hbar }$$

For the pristine graphene sample, propagating plasmon mode only exists in the armchair ribbon, which shows characteristic metallic properties. Because of the chirality of the wave functions, plasmon mode in armchair ribbon is unable to decay into particle–hole pairs. While for pristine graphene ribbon of zigzag type, plasmon mode decays into particle–hole pairs easily in a wide spectral range, thus no plasmon mode can be supported. For doped graphene ribbons, plasmon dispersion properties are similar to that of semiconductor nanowires, the dispersion law is defined by the proportion of $q_{x}^{3/2}\sqrt{-\ln(q_{x}W)}$, in which the index is related with the doping condition. Furthermore, the single particle spectrum around *E_F_* is shown by the curve of excitations between particle and hole, beyond the curve, the dispersion of plasmon mode comes as $q_{x}\sqrt{-\ln(q_{x}W)}$, thus the plasmons in doped graphene nanoribbons are similar to that of nanowires.

Plasmons in two dimensional materials have been experimentally observed in many ways, which can be either direct or indirect. For example, the traditional optical measurements, light scattering methods as scattering‐type scanning near‐field optical microscopy (scattering‐type SNOM), angle‐resolved photoemission spectroscopy (ARPES), electron energy loss spectroscopy (EELS), and scanning tunneling spectroscopy (STS).^12^, ^13^, ^60^, ^61^, ^62^ By those techniques, plasmons on free standing graphene sheets and on epitaxial graphene samples are well analyzed, furthermore, many articles reported that biased graphene can also support SPPs with wavelengths much smaller than the wavelength of the incident wave. For example, an incident beam with the frequency of 30 THz can excite SPPs with the wavelength of 200nm.

The Dissipation effect of SPPs can be derived from the propagation length in the unit of SPPs wavelength. The curves in Figure 2b show the calculated propagation length of gold and biased graphene. The propagation length meets well with the SPPs wavelength of biased graphene which is in the infrared region as shown in the black curve and red curve, but the calculation results which come from the experimental data are lower than that derived from the theoretical data. A slightly improved propagation lengths as the red curve shows, which is calculated based on the theoretical data of graphene under a bias voltage of 71 V, but not longer than three SPP wavelengths, since the interactions of electrons and electrons are concluded. As the fabrication technique of graphene improves, better clean monolayer graphene with lower dissipation and longer propagation length can be utilized to manipulate SPPs at a micrometer scale and one atom thickness, in the infrared spectrum region. Biased graphene can be a better candidate to support surface plasmons, with larger kinetic inductance than that of unbiased sample. This energy loss effect is also analyzed by the high‐resolution angle‐resolved reflection electron‐energy‐loss spectrometer (HREELS) as shown in Figure 2c. In the experiment, the low‐energy (about 20.29 eV) electrons are backscattered from graphene on a SiC substrate and form several energy loss peaks on the HREELS spectral, those peaks increase and disperse strongly which depends on the increasing of momentum transfer *q* with the direction parallel to the surface of graphene. All curves show two nondispersing low loss peaks which are marked as P1 with the energy of 67 meV and P2 of 159 meV, P1 comes from the low‐energy π plasmon, which is reported in the previous research on graphitic surface, while the physics origin of P2 peak is Fuchs‐Kliewer (FK) optical phonons supported by both graphitic structures on SiC substrate and the substrate surface itself. This two peaks are combined by the original background and sheet plasmon peaks which make them hard to be observed, while they emerges as the steep intensity drops induced by the increasing of off‐specular angle. Notably, the plasmons with low energy have almost identical dispersion at small *q*, however the dispersion relation is different from that of *q* ^1/2^ dispersion with increasing *q*. In two dimensional graphene sheet, the data falls below the *q* ^1/2^ curve for the wave number (*q*/*k_F_*) between 0.1 and 0.5, as shown in Figure 2d, additionally, the loss of plasmon energy is strongly relative to the Fermi energy *E_F_* for the 2D surface state.

Under the driving force of incident electromagnetic field, electrons are collectively pulled from their balance position and screen outer electric field, however, they tend to be moved too strong and then they are forced back by the Coulomb force. This movement is able to continue and form an oscillating system, in which the restoring force comes from the summation of electric field created by all the electrons in the material. The dispersion relation of two dimensional plasmon can be well calculated at the long wavelength (q << k_F_). In fact, those collective charge density oscillations of the electron gas propagating through the medium with specific dispersion relation can also interact with other quasiparticles. For example surface plasmon polaritons which are the bound states of electrons or holes with collective excitations of a many‐particle system, and induce strong self‐energy effects. It has been theoretically predicted that those composite quasiparticles named plasmarons, which consist of the carrier charges and plasmons in the material. The energy band of plasmarons can be observed by high‐resolution ARPES, which exist in the graphene sheet on SiC substrate.^63^ As shown in Figure 2e, the Dirac energy spectrum of quasi‐freestanding graphene is shown on the left panel, and it is in a non‐interacting picture, the momentum has two components as *k_x_* and *k_y_*, while E_D_ presents the energy of the Dirac point. The right panel shows the Dirac spectrum considering interactions of electrons, and it illustrates the characteristic reconstructed Dirac crossing. Measured by ARPES, the energy band structure of n‐doped graphene on a SiC substrate is presented in the middle, there are four bands crossing at the Dirac energy (E_D_) instead of two, which indicates states around E_D_ from a single point recombine and change to the shape like a diamond. The strong reconstruction of the massless Dirac Fermions (MDFs) chiral spectrum near the Dirac point of doped graphene supports that the self‐energy comes from the interactions between one charge carrier and the two dimensional collective electron gas in monolayer graphene.

### 2.3 Strong Coupling Between 2D Excitons and Plasmons

LTMDCs show promising characters to support strong coupling effects between light and matter, because of their advanced electronic structures and optical properties as mentioned above. Strong coupling effect is achieved when the relaxation process changes to new path, which comes from the coherent energy transfer between the transition of exciton and resonant optical cavity. This process further induces several light‐matter hybrid states and new quasi‐particles separated by the Rabi splitting which are named as exciton polaritons.^64^, ^65^ Notably, the properties of polaritons are unique, which include small effective mass, fast propagation speed, long‐range coherence and strong matter interaction abilities, make LTMDCs be suitable for optoelectronics, sensing and studies of ultrafast process.^66^, ^67^ The strong interactions of photons, plasmons and excitons inspire many fascinating studies such as Bose−Einstein condensation, efficient charge transfer, and active tuning of material phase transition.^68^, ^69^, ^70^ As the researches for hybrid nanomaterials of 2D materials and plasmonic nanostructures develop fast, the study of strong light‐matter coupling effect attracts growing interest. A team successfully fabricated a dielectric microcavity structure which is comprised of a MoS_2_ layer inside and achieved strong coupling effect between light and matter.^71^ In this heterostructure, strong coupling between MoS_2_ excitons and the cavity photons is achieved and a Rabi splitting is observed by an angle‐resolved reflectivity and PL spectral method at room temperature.

The advance of metallic plasmonic system is strong filed enhancement in a subwavelength scale, as SPPs harvest the energy of light in high efficiency. The coupling effects between excitons and plasmons are widely found in many hybrid nanomaterial systems such as plasmonic nanoparticles and structures hybridized with J‐Aggregate or quantum dots.^72^, ^73^, ^74^, ^75^, ^76^, ^77^ For example, significant Rabi splitting is observed which reaches to hundreds of meV and reveals strong coupling effect between excitons and plasmons. In the Rabi splitting process, coupling strength quantified as *ℏ*ΩR, strongly depends on the electric field intensity *E* in the cavity and the dipole moment *d* of exciton transition. At the condition of scalar product of *E* and *d* reaches to the maximum, the coupling effect becomes the strongest, while with the minimized loss of energy. Thus, periodic plasmonic structures which are comprised of separated metallic nanostructures, are of special interest in the field of strong coupling studies. In those systems, LSPRs are achieved and the near electric field are highly enhanced in each structure unit. In the whole arrangement of periodic structures, due to the interaction between different modes of LSPRs and individual diffractive orders, sharp resonances are highly supported.^78^ In this situation, periodic plasmonic structures show unique filed enhancement efficiency and resonance coupling efficiency with high quality factor, which makes them a suitable candidate to achieve coherent coupling between excitons and plasmons in the system of hybrid nanomaterials.

The experimental observation of strong coupling effect between the 2D material excitons and plasmons of metallic nanostructures is of growing interest.^79^ The existence of three types of resonances in the hybrid structure is proved, which includes excitons, lattice resonances, and LSPRs. A coupled oscillator model is demonstrated to explain the coupling resonances and fit the dispersion curves, which includes five oscillators as A/B excitons, diffractive modes of (+1,0) and (−1,0) orders, LSPR supported by nanodisks. Measuring the reflectance spectra derived from the bare MoS_2_ monolayer, excitonic energies of A and B are calculated, while the lattice dispersion and LSPR term are fitting parameters to take the MoS_2_ induced spectral shift into consideration. In this model, the coupling between A, B excitons and between two diffractive modes are ignored, and the coupling of other resonances are described as fitting parameters. The coupling between lattice diffraction and LSPR becomes stronger with increased disk diameter, while the exciton‐LSPR maximum coupling strength is achieved when the resonances of LSPR and the A exciton settle on the same spectral wavelength. The coupling strength between exciton and lattice diffraction mode is the weakest, and the coupling between exciton and plasmon is dominant in the MoS_2_/Ag nanodisks hybrid structure, and this strong coupling effect is dependent on the LSPR mostly. Notably, a coherent coupling exists in a large scale, which breaks the localized region of LSPRs, and enhances the coupling effect of various resonances.

**Figure 3:**
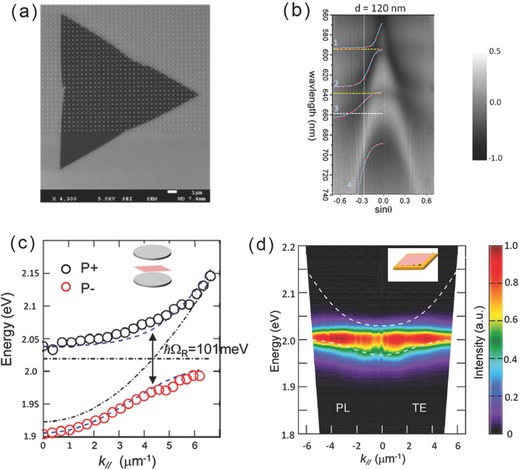
a) SEM image of MoS_2_/silver nanodisk hybrid structure on a Si/SiO_2_ substrate. b) Angle‐resolved differential reflectance spectra of MoS_2_/silver nanodisk hybrid structure, solid lines are multi‐oscillators model fitted results. (a,b) Reproduced with permission.^[79]^ Copyright 2016, ACS. c) Peak transmission energies as a function of in‐plane angular momentum for MoS_2_/FP cavity hybrid structure, the Rabi splitting of 101meV exist between P+ and P‐ branch. (Inset) the schematic view of the hybrid structure. d) Photoluminescence dispersion of MoS_2_/plasmonic hole array hybrid structure. (Inset) the schematic view of the hybrid structure.(c,d) Reproduced with permission.^[27]^ Copyright 2016, ACS.

The SEM image of the MoS_2_/Ag nanodisks hybrid structure on Si/SiO_2_ substrate is shown in **Figure** **3**a, the period of silver nanodisk array is 460 nm while nanodisk diameters are various to support the LSPR wavelengths in a wide range to resonate with the MoS_2_ exciton (590–650 nm). The coupling of five kinds of oscillators is shown by angle‐resolved reflectance spectrum of MoS_2_/Ag nanodisks hybrid structure (Figure 3b), which is comprised of five polariton branches with different excitonic and plasmonic fractions. By analyzing spectral branches, the coupling properties of hybrid structures can be concluded as follows: First, the interactions between excitons and plasmons of each polariton branches can be tuned by the resonance frequency. Second, the polariton dispersion condition is significantly dependent on different lattice designs and the corresponding lattice diffraction resonances. Because of the non‐propagating and flat dispersion nature of LSPRs, the effective masses of excitons also show the similar flat dispersion properties of which the magnitudes are related with different lattice modes. Finally, the intensity of coupling strength can be modulated by the splitting numbers between the two polariton branches. The strongest coupling between exciton and plasmon is achieved when LSPRs become in resonance with MoS_2_ excitons and detuned lattice resonances.

However, the coupling condition in the above system is complicated because there are two kinds of excitons in MoS_2_ induced by the SOC effect, which simultaneously interact with diffraction modes. Monolayer WS_2_ exhibits single sharp absorption band and shows a sharp PL peak at 2.016 eV. A team demonstrated a WS_2_/Ag Fabry−Pérot (F‐P) cavity hybrid structure which achieved strong coupling between excitons and plasmons at room temperature.^27^ Because of the strong absorption efficiency of F‐P cavity and higher excitonic efficiency of WS_2_, a significant Rabi splitting of 101 meV is observed (Figure 3c). For hybrid structure of WS_2_ and plasmonic arrays, the coupling becomes weaker but still achieves a 60 meV Rabi splitting, and reveals new PL properties (Figure 3d). A coupled oscillator model is demonstrated as follows to fit the experimental spectra:

(5) $$\left[\begin{array}{cc} E_{ph\left(k∥\right)} & V\\ V & E_{ex} \end{array}\right]\;\left(\begin{array}{c} \alpha \\ \beta \end{array}\right)\;\;=\;\;E_{pol}\left(k_{∥}\right)\;\left(\begin{array}{c} \alpha \\ \beta \end{array}\right)$$

In the function, *E_ph_* (*k*_ǁ_) and *E_ex_* stand for the energy of empty photonic mode and exciton, *V* is the interaction potential between photonic mode and exciton, and is equal to *ℏ*ΩR/2. *E_pol_* (*k*_ǁ_) denotes the eigenvalues of splitting branches and the value depends on the in‐plane component of the incident light momentum *k*_ǁ_. |α|^2^ and |β|^2^ are derived from the Hopfield coefficients, which correspond to the photonic and excitonic contents of the polaritonic states. In a certain system, the dispersion relation of plasmonic modes can be derived, for example, the dispersion function in plasmonic hole array follows the form as:

(6) $$\left|{\vec{\;k}}_{spp}\right|\;=\;\left|\vec{\;i}\left(m\frac{2\pi }{P}\right)\;\;+\;\vec{\;j}\left(n\frac{2\pi }{P}\;+\;k_{∥}\right)\;\right|$$

In which, *P* is the lattice period, while (*m*, *n*) corresponds to the scattering orders of SP modes. Considering |*k*_∥_| ≤ 2π/*P*, the dispersion of TM (0, ±1) mode can be written as:

(7) $$\begin{array}{c} \omega \;=\;\frac{c}{n_{sp}}\left(k_{∥}\;+\;\frac{2\pi }{P}\right)\\ \omega \;=\;-\frac{c}{n_{sp}}\left(k_{∥}\;-\;\frac{2\pi }{P}\right) \end{array}$$

By applying this multi‐oscillators model into the strong coupling hybrid structures, dispersion curves can be well fitted in transmission, reflection, and emission spectral. In conclusion, strong light−matter coupling of hybrid structure inspires the designs of ultrathin and high efficient plasmonic polariton devices, enables more methods on chemical reaction rate control and chemical bond vibration studies. But, the interaction properties of excitons, photons, plasmons and phonons in hybrid structures with growing complexity stay unclear, and need to be analyzed in more detail.

## 3 Plasmonic Tuning of 2D Nanomaterials

The properties of plasmons in 2D nanomaterials show strong dependence on the material band structure, which can be influenced by chemical and electric doping effects. Also, constructing 2D nanomaterials into specific patterns and inducing defects can also modulate the plasmonic properties, which come from the boundary condition. More importantly, 2D nanomaterials hybridized with metal nanostructures give us new ideas for the plasmonic modulation methods since the plasmonic tuning techniques of different metal nanostructures have been profoundly researched and show great possibilities, along with advantage of the strong field enhancement effect.

### 3.1 Plasmonic Tuning of Doped 2D Nanomaterials

At specific wavelength, the incident electromagnetic wave becomes resonant with the surface plasmon and can be trapped in a subwavelength volume, leading to a strong field enhancement. For doped graphene, the properties of surface plasmon are strongly linked with the in‐plane conductivity, which include the intraband and interband transitions of hot electron hole pairs, and are mainly dependent on the parallel wave vector and wave frequency. Within the RPA method in the local limit, the light matter conditions can be expressed as:

(8) $$\begin{array}{l} \sigma \left(\omega \right)\;=\;\frac{e^{2}E_{F}}{\pi \hbar^{2}}\frac{i}{\omega \;+\;i\tau^{-1}}\\ \;\;\;\;\;\;\;\;\;\;+\;\frac{e^{2}}{4\hbar }\left[\theta \left(\hbar \omega \;-\;2E_{F}\right)\;+\;\frac{i}{\pi }\log\left|\frac{\hbar \omega \;-\;2E_{F}}{\hbar \omega \;+\;2E_{F}}\right|\right] \end{array}$$

**Figure 4:**
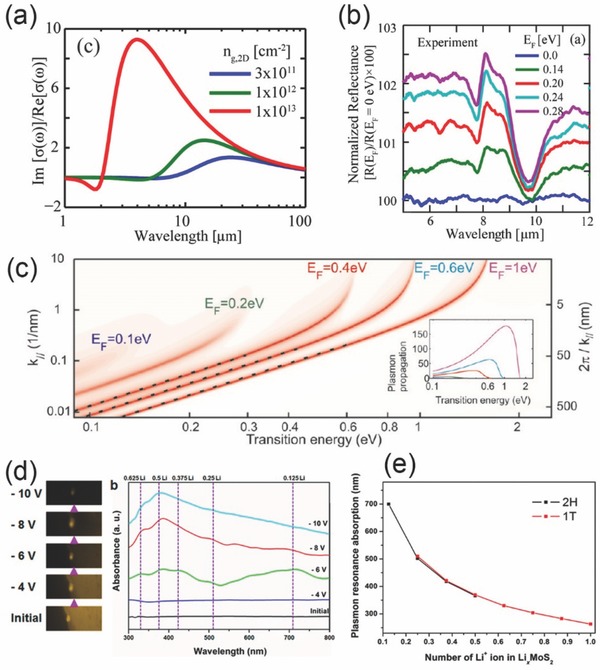
a) Plasmonic response of graphene with different carrier density. b) Graphene IR reflection spectra with various sheet carrier density. a,b) Reproduced with permission.^[80]^ Copyright 2015, De Gruyter. c) Plasmon dispersion relation in doped graphene. (Inset) the propagation decay in the unit of SPP wavelength. Reproduced with permission.^[81]^ Copyright 2011, ACS. d) Left panel shows PL images of monolayer drop‐casted MoS_2_, the right shows the absorption spectrum of 2D nanoflakes under electrochemical force control. e) Schematics of plasmon resonance peak positions as the function of Li+ ions number in 2D MoS_2_ nanoflakes for 2H and 1T phases. d,e) Reproduced with permission.^[82]^ Copyright 2015, ACS.

The first term of equation shows the intraband transitions induced Drude mode response of graphene, as shown in **Figure** **4**a in the mid infrared region, the intraband response is the dominant factor for the spectrum and makes graphene plasmon exist in a board mid infrared region.^80^, ^81^ Notably, τ is the finite relaxation time, *T* is the temperature, and *k_B_* presents the Boltzmann constant. Since the Fermi energy follows the function as $E_{F}\;=\;\frac{hv_{F}}{2}\sqrt{\frac{n_{g,\;2D}}{\pi }}$, which indicates the strong dependency of carrier concentration in graphene. Additionally, the higher carrier density *n*_g,2D_ in graphene will induce plasmonic response to the near infrared region, which broadens the resonant wavelength of graphene efficiently. The second term arises from interband transitions, and plays dominant role in the visible and near infrared region, since the loss is dramatic above 2*E_F_* energy. As mentioned above, the optical conductance is a constant in the spectral region. The normalized infrared reflection spectra of monolayer graphene with different carrier density are shown in Figure 4b. When the carrier concentration becomes higher, the reflectivity of monolayer graphene is also increased, which shows more properties like metal. However, if *E_F_* > ω which is on the condition of high enough doping intensity, surface plasmons will be able to propagate within the highly doped graphene sheet with the wave vector as:

(9) $$k_{sp}\;\approx \;\left(\frac{h^{2}}{16\pi^{2}e^{2}E_{F}}\right)\;\left(\in \;+\;1\right)\omega \left(\omega \;+\;i/\tau \right)$$

This function shows characteristic quadratic dispersion relation of graphene, meeting well with the theoretical calculation of 2D electrons. As shown in Figure 4c, this expression also indicates that the SPPs of graphene show strong dependence on the doping effect, furthermore, the wavelength of graphene SPs can be expressed by the free space wavelength of light, and are deduced as *λ_sp_* ≈ [4α/(ε + 1) (2*πE_F_*/(*hω*)]*λ_0_*. Additionally, the wave vector with the direction out of the plane shows the mode concentration within the subwavelength thickness, which is approximately *λ_sp_*/2π in *z* direction. While the propagation distance within the doped graphene plane is much longer, decided by the imaginary part of *k_sp_*, the SPP distance as the function of transition energy can be well modulated by different doping effects (the inset of Figure 4c), and the maximum of more than 100*λ_sp_* can be reached. At high energy, the interband transition will become dominant since the plasmons are able to generate electron hole pairs with enough energy. The shape of curves tends to be narrower with higher Fermi energy, since the relaxation time τ increases as the function of incidental light frequency ω.

Apart from the unshaped graphene, doping effect can also be utilized to tune the plasmons of monolayer semiconductors such as MoS_2_.^82^ The plasmonic properties of monolayer MoS_2_ mixed with lithium ion can be investigated via optical properties as absorption or light emission in the spectral region of ultraviolent to visible light. Researchers analyzed the plasmon resonances of monolayer MoS_2_ nanoflakes mixed by Li^+^ ion with electrochemical methods and successfully found the plasmon resonances in the visible and near ultraviolent region. The plasmon resonances of the highly doped monolayer MoS_2_ nanoflakes after the intercalation show two peaks corresponding to different crystal phase. Figure 4d shows the PL images of two dimensional monolayer MoS_2_ without intercalation and with different intercalation intensity by controlling applied voltages. The light emission of monolayer MoS_2_ nanoflakes shown in yellow is quenched with decreasing intercalating voltages (initial state 0 to –10V) and becomes negligible at –10 V. While the absorption spectra of the MoS_2_ sample shows that the doping efficiency of Li+ ions is not high for MoS_2_, since no distinctive peaks exist for –4 V intercalation. Under continuously decreased voltage, more free electrons generate in the nanoflakes with both 2H and 1T phases further form several absorption peaks at −6 V, and the resonance position meets well with the theoretical prediction as shown in Figure 4e. However, under lower intercalating voltages, the arrangement of monolayer MoS_2_ reminds only 1T phase. This significant phase conversion from 2H to 1T enables efficient plasmonic modulation over a wide spectral range. There are more methods of carrier density control such as compacting MoS_2_ sheet into a FET device with different gate bias voltages, gas physisorption, and chemical doping, which provide more choices for plasmonic tuning of unstructured 2D nanomaterials.^83^, ^84^

### 3.2 Plasmonic Tuning of 2D Nanomaterials Plasmon by Structure Control

**Figure 5:**
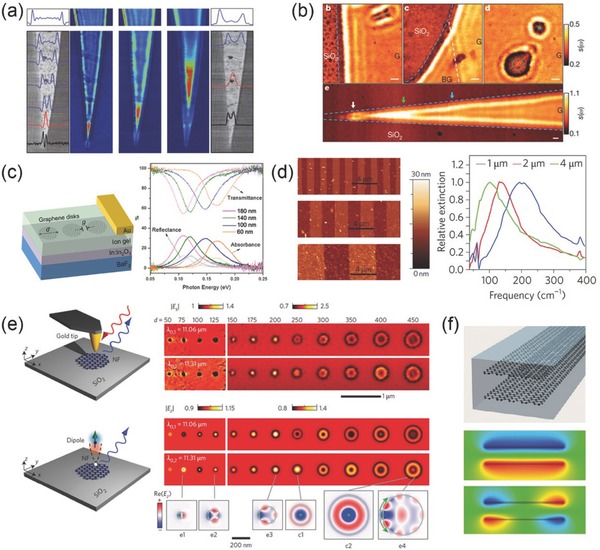
a) Near‐field images of resonant localized mode on tapered graphene ribbon taken with different imaging wavelength. Reproduced with permission.^[86]^ Copyright 2012, Nature Publishing Group. b) Photograph of graphene in IR spectrum region, the wave pattern shows significant boundary and defect sensitivity. Reproduced with permission.^[87]^ Copyright 2012, Nature Publishing Group. c) Left: structure of graphene disks array device, right: corresponding transition and reflection spectra with changing disk diameter. Reproduced with permission.^[89]^ Copyright 2014, ACS. d) Left: AFM image of different widths of graphene ribbon arrays, right: Localized plasmonic resonance induced extinction spectral in monolayer graphene ribbons. Reproduced with permission.^[91]^ Copyright 2011, Nature Publishing Group. e) Experiment and simulation results of localized plasmonic resonance modes analysis in graphene disks on SiO_2_ substrate. Reproduced with permission.^[90]^ Copyright 2016, Nature Publishing Group. f) Upper: graphene ribbon pairs, lower: near field image of two plasmonic resonance mode. Reproduced with permission.^[92]^ Copyright 2011, ACS.

The structural modulation of 2D nanomaterials plasmon, especially graphene plasmon, leads an approach to modulating several characteristics.^85^ A team found a way to tune the plasmon resonance wavelength to a large degree by slightly changing the excitation wavelength.^86^ They investigated the plasmon resonances of graphene nanoribbons on the substrate of SiC with different excitation wavelengths by the method of scattering‐type SNOM. Scattering‐type SNOM normally comprises an atomic force microscope (AFM) in which the metallized tip is illuminated by a focused infrared laser beam, and the backscattered radiation is recorded simultaneously by the topography method of yielding nanoscale resolved infrared near‐field images. So this technique makes spectroscopy and infrared nano‐imaging of the materials without fabricating specialized periodic structures become possible. The experimental results of near‐field image (up image in **Figure** **5**a) show the dependence of the fringe space on the dielectric constant of the substrate SiC that the spacing of the fringes decreases dramatically with increasing dielectric constant. And when the relation between the excitation wavelength and the dielectric constant is taken into consideration, it shows that the spacing of the fringes increases greatly with the excitation wavelength, respectively. The observation results are consistent with the equation λ_p_ ≈ 4*λ_0_αE_F_*/*E_P_*(1 + *ε_r_*), in which *E_F_* and *E_P_* represent the Fermi energy and the plasmon energy respectively, λ_0_ represents the excitation wavelength, and *ε_r_* is the substrate permittivity. In this way it is speculated that the carrier density in narrow ribbons is greater than that in larger one. When the width reaches values smaller than the plasmon wavelength *λ_p_*, the near‐field image (below image in Figure 5a) shows a clear shift of the localized modes to a wider part of the ribbons for increasing *λ_p_*, and the topography images of the ribbons obtained by AFM (image in grayscale in Figure 5a) shows the change of the hotspot location. The observation above reveals the change of the localized modes at different excitation wavelengths and presents a new way to control the plasmon behavior in graphene ribbons by tuning the excitation wavelength.

Apart from controlling the excitation wavelength to tune the plasmonic characteristics, through introducing defects into the nanomaterials we can also observe similar results. Using a scattering‐type SNOM, it is possible to experimentally observe the plasmonic characteristics of graphene ribbon after a defects introducing process.^87^ The images of infrared amplitude of graphene ribbons on the substrate of SiO_2_ (Figure 5b) show the characteristic interference pattern (blue dashed lines) and defects (green dashed lines and green dot). This reveals the periodic oscillations of signal along the graphene edge and shows that the line defect can produce the pattern that is different with the long gated one, those fringes are produced in both sides of the boundary. Therefore, plasmon resonances pattern can be effectively changed by introducing defects.

Besides, the modulation of sample structures of graphene can provide distinctive characteristics compared to the traditional nanoribbons. For instance, the structures of disk arrays and nanoribbon arrays of graphene bring novel features.^88^, ^89^ A device is designed (left image in Figure 5c) to promote the optical absorption at visible and infrared wavelengths in graphene nanodisk arrays. In this device, with the method of electron‐beam lithography, the monolayer graphene is configured to specific structures on In−In_2_O_3_/BaF_2_ substrate. And then ion gel is spin‐coated on top of the graphene nanostructure with Au gate contact deposited above. By FTIR measurements of transmittance, reflectance and absorbance results (right image in Figure 5c) exhibit prominent resonances which associate with the excitation of plasmons in the nanodisk arrays. As the Fermi energy *E_F_* changes from 0.2 eV to 0.8 eV, both the reflectance and the absorbance increase while the transmittance decreases. This phenomenon coincides with the theoretical results brought by the coupling between nanodisks, as the peaks in the spectra of nanodisk arrays can match the calculated plasmon modes energies of the single nanodisks. In this way, by taking advantages of graphene nanodisks, the aim to promote the absorption efficiency in the infrared region of the spectrum can be achieved. The result that the efficiency can be increased from less than 3% to 30% in the infrared region makes nanopatterned graphene promising for practical utilizations of infrared electro‐optic devices.

Similarly, a device which consists of graphene nanoribbon arrays is reported. In this experiment the plasmon resonances and light‐plasmon coupling in graphene micro‐ribbon arrays were studied by Fourier transform infrared spectroscopy. The images obtained by AFM reveal that the nanostructure of the graphene nanoribbon arrays agree with the theoretical calculation results. And the results (right image in Figure 5d) of the transmission spectra show that the samples with different micro‐ribbon widths red‐shift when the ribbon width decreases. For infrared absorption from doped charge carriers is related to *–*△*T*/*T_cnp,_* the decrease of transmission through graphene micro‐ribbon arrays, where *T_cnp,_* is the transmission coefficient at CNP and △*T* = *T–T_cnp_*. This nanostructure of graphene nanoribbon arrays has similar effect in promoting the graphene efficiency in absorption in infrared regime, which can be applied in electro‐optic devices.

Together with the experimental results and the great potential applications in several directions, the theory of the structural modulation's effect on the 2D nanomaterials plasmon is attracting great attention. Researchers investigated the graphene plasmon modes in graphene disk nano‐resonators on the SiO_2_ substrate.^90^, ^91^ They analyzed a set of disks at two different illumination wavelengths with the diameter d increasing from 50 to 450 nm. The results (up image in Figure 5e) show the near‐field features of small disks, which exhibit a bright ring and a dark center. For medium disks, they have a dark ring and a bright center. As for the larger disks, they have both a dark ring and a dark center. This phenomenon changes little with the increase of the illumination wavelengths, and shows the disk size induced influence on the graphene plasmon modes. To further investigate this effect, they also analyzed the experimental results by numerical electromagnetic calculations. In the stimulation, a dipole source is the regarded as the illuminating tip in the experiment. By plotting the near field below the dipole, the simulated near‐field images (image below in Figure 5e) were obtained and generally matched the experiment results. Besides, when the dipole is located at the center of the brightest disk, a completely different plasmon mode appears in the dipole because the field oscillations are strongly confined to the edge of the disk and propagate along it. This indicates that tip can efficiently probe both plasmonic sheet and edge mode in graphene, and through structural modulation the probed plasmon modes can be tuned.

Apart from the structural modulation above, the strong interlamination coupling can also affect the plasmonic properties in graphene.^92^, ^93^ It is found that the plasmon modes of the interacting graphene nanoribbons are different from that of a single nanoribbon. The electric near‐field results (images below in Figure 5f) show that because of the electrostatic scaling law, the interaction between plasmons in coplanar ribbon pairs gives rise to the hybridized state, and converges to the modes of the ribbon of double width at zero separation. This gives rise to the two lowest‐energy bands, and the near fields of these modes basically show binding and anti‐binding combinations of monopoles, without nodes in the induced charge of each ribbon. In this way, the guided plasmons in paired graphene ribbons exhibit great difference in distribution of modes comparing that in individual ones. 2D plasmons in doped graphene show larger confinement and longer lifetime than that in noble‐metal. Additionally, with the introduction of the interacting paired graphene nanoribbons, the potential of the graphene plasmon circuits for future plasmon devices becomes clearer, for instance in infrared sensing and optical signal processing applications.

### 3.3 Plasmonic Tuning of Hybrid 2D Nanomaterials

**Figure 6:**
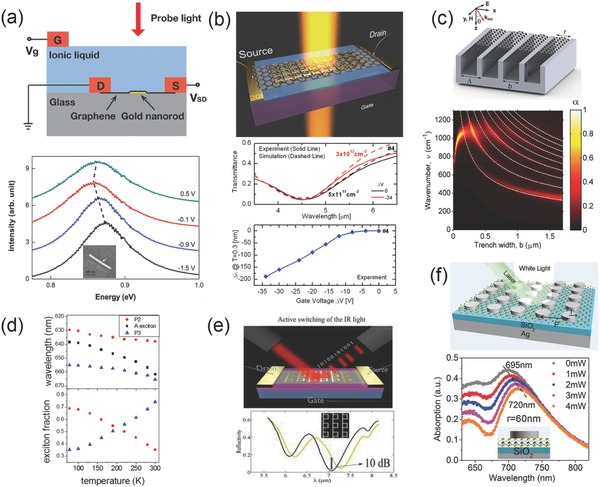
a) Top: illustration of graphene‐gold nanorod hybrid structure, bottom: Rayleigh scattering spectra of hybrid structure at different gate voltage. (Inset) SEM image of nanorod on graphene sample. Reproduced with permission.^[103]^ Copyright 2012, ACS. b) Top: schematic illustration of electrically tunable graphene‐bowtie antennas hybrid structure, middle: changes of plasmonic resonance damping under applied gate voltage, bottom: plasmonic resonance width changing as the function of gate voltage. Reproduced with permission.^[104]^ Copyright 2012, ACS. c) Upper: illustration of graphene ribbon array hybridized with metal gratings structure, lower: absorption mapping of the structure with changing trench width. Reproduced with permission.^[101]^ Copyright 2015, ACS. d) The spectral position of the A exciton and two polariton branches for both pristine MoS_2_ and nanodisk‐MoS_2_ hybrid structure as the function of temperature. Reproduced with permission.^[79]^ Copyright 2016, ACS. e) Upper: schematic view of Fano‐resonant structure hybrid gated graphene structure, lower: the reflectance spectrum under electrical switching. Reproduced with permission.^[99]^ Copyright 2015, ACS. f) Upper: illustration of Ag disk‐MoS_2_ hybrid structure under laser illumination, lower: changing of localized plasmon shifting as the laser power changes. Reproduced with permission.^[96]^

Plasmonic resonance can be actively tuned by electronic doping as mentioned above, however the modulation range is mainly settled in terahertz and MIR region, the plasmonic tuning of optical frequency remains a challenge. Plasmonics of metallic nanostructures and nanoparticles are deeply researched and it is proved that plasmonic resonances in metallic nanostructures and 2D materials interact with each other strongly, if metallic structures are configured near 2D materials and form a hybridized heterostructure.^94^, ^95^, ^96^, ^97^, ^98^ By utilizing the known properties of specific metallic structures pattern and unknown hybridization possibilities, quantities of heterostructure are designed and show excellent tuning abilities over a wide spectral range.^99^, ^100^, ^101^, ^102^ A hybridization of graphene and gold nanorod device is presented in **Figure** **6**a, which enables active plasmonic tuning of both resonance frequency and quality factor in the near infrared region.^103^ In this study, both simulation and experiment results confirm that the quality factor of plasmonic tuning effect by electrical gating method is 28%. The most contribution comes from the ends of the rod, where two plasmonic hot points exist. Simulation proved that the in‐plane electric field near the spots is enhanced intensively, and holds a large proportion (14%) of integrated field intensity among the total graphene sheet area. This little proportion of area only contains tens of electrons induced by gating process, but contributes to a significant charge carrier concentration variation of 8 × 10^12^ cm^−2^, shifts *E_F_* to a 0.5 eV level, and further blocks the interband transitions in graphene. As a result, the scattering intensity sensitivity to the hot carriers number exists at the hot spot can be very high (0.07% per carrier), making it possible for a single electron observation form scattering intensity spectrum of this hybrid graphene gold nanorod device. The strong interaction between graphene and metallic plasmon also offers an efficient method to control the plasmon resonances at optical frequency, which can be useful for more practical applications.

Bowtie structures can also support strong electrical hot spot, since the sharp corner will concentrate electrons in high intensity.^104^ A design of a FET device of Bowtie array hybridized large area graphene is reported which has highly tunable carrier concentration under electrostatic gating, the plasmonic structures fabricated on graphene enhance the interaction of the incident optical field with the graphene sheet, and the impact of graphene is much stronger at mid‐infrared wavelengths. Figure 6b is the schematic illustration of the experimental structure for voltage‐controlled optical transmission measurement, which indicates the plasmonic damping in the hybrid structure. The image below shows the gate voltage's effect on the plasmonic resonance in which the experiment results match well with the simulation results to a large degree in measuring the transmission spectra. By introducing hot electrical spots, graphene can be used to electrically control the damping of plasmonic resonances in the mid‐infrared spectral region. Better optimized design will be explored to achieve efficient damping and tuning of plasmonic resonances.

Because of the atomic thickness of monolayer graphene, it is difficult for single layer graphene ribbons to achieve efficient absorption. A hybrid system of graphene ribbon arrays are further investigated and find that the localized resonances in metal gratings can couple with the plasmonic resonances in graphene ribbons and increase the absorption to a large degree.^101^ The schematic view in Figure 6c shows the hybrid structure of plasmonic metallic grating and graphene ribbons under plane wave illumination. *Λ*, *h*, and *b* are the period, height, and trench width of the grating as well as the graphene ribbon array, and *r* is the width of the ribbon. The image below illustrates the contour plot of the absorption when the incidence angle is fixed at 50° with varying trench widths while the ribbon width remains the same as the trench width (*r = b*). It exhibits additional bright bands corresponding to the even‐order plasmons. These findings may facilitate the design of optoelectronic devices and metamaterials structures based on hybrid nanostructures and graphene. Several types of modulators are analyzed and show excellent modulating functions in the telecom wave range at low gating voltages for a very small active device area. Those properties are nearly as the same as traditional waveguide modulators which are silicon‐based, but the ultra‐thin characteristic makes them promising for compact device, and further makes them possible for realization of graphene‐based plasmonic modulators for fast optical communications.^105^, ^106^

The exciton‐plasmon coupling effects give more insight about the tuning of plasmons in hybrid 2D nanomaterials, which have been discussed before and enable us to actively modulate plasmonic properties of 2D nanomaterials via temperature control.^79^, ^96^, ^107^ Through MoS_2_ excitons, localized surface plasmon resonances (LSPRs) of individual silver nanodisks and plasmonic lattice resonances of the nanodisk array these three types of resonances, novel 2D plasmonic polaritonic devices can be realized. Figure 6d shows the temperature dependence of exciton‐plasmon polaritons. The up one shows the relation between the wavelength of the A exciton and two polariton branches and the temperature. It is presented that at low temperature the energy of the branch 2 is closer to the A exciton compared to polariton branch 3. And as the temperature increases, branch 2 red‐shifts faster. While at high temperature, the energy of the branch 3 is closer to the A exciton on the contrast. And the image below shows the calculation results of the exciton fractions. Polariton branch 2 contains 0.7 exciton fraction at 77 K and is more matter‐like and temperature dependent, while at the room temperature, this proportion drops to 0.3, and it becomes more light‐like and temperature independent. For polariton branch 3, on the other hand, the exciton fraction increases from 0.35 to 0.75 from 77 K to room temperature. Those results further illustrate that the physical properties of the light‐matter interactions in emitter‐coupled plasmonic lattices can be tailored by tuning the relative fractions of different resonances with different features.

Active tuning methods of plasmons are of more practical interest, for now the working spectral region is mainly in infrared light in graphene. As is known to all that the optical response of graphene is rather weak, the method to increase its absorption in the infrared part of the spectrum has become the key point for its optoelectronic applications.^99^, ^108^ Through electric gating the easy approach to switching the light can be taken. In Figure 6e, the upper image shows the structure of the device. Through the hybridization of Fano‐resonance structure and the gated graphene structure, the optical properties change a lot. The image below shows the reflectivity spectrum of the hybrid structure under the electric gating. It shows that the ultrafast response time is accomplished due to rapid injection of charge carriers into graphene, while large modulation depth (about 10 dB) in reflection is achieved by the metasurface design. The designed metasurface here is promising for light modulation by either electrostatic or chemical doping. Thus applications for the infrared absorption and spectral response control are attractive. More designs about the active control of plasmon‐exciton coupling in hybrid nanostructures are reported as shown in Figure 6f which is the schematic of the MoS_2_‐Ag hybrid nanostructure and coupled oscillators model for intuitive explanation. The MoS_2_‐Ag hybrid nanostructures are fabricated by designed Ag‐disk structures onto the surface of MoS_2_ monolayers, which have strong response to the incident light. The image below shows the absorption spectra of MoS_2_‐Ag hybrids with disk radius of 60 nm, where the LSP resonance red‐shifts with the increasing of the laser power. Total shifting of 25 nm can be obtained as the laser power increased. This work demonstrates that the plasmonic response of MoS_2_ monolayers can be effectively modified by the photoexcited excitons, which play a significant role in controlling surface plasmon resonances in MoS_2_‐Ag hybrid nanostructures. The capability of active control of exciton–plasmon coupling is able to provide new opportunities for high‐performance and ultra‐thin optoelectronics devices.

## 4 Plasmon‐Induced Applications of 2D Nanomaterials

Some 2D nanomaterials with atomic thin thickness show unique optical properties, such as direct band gap light emission, valley polarized PL and distinctive Raman signals. However, the monolayer regime of 2D nanomaterials provides a significant challenge for weak light‐matter interaction, which limits their applications in light‐emitting and optoelectronic devices. 2D nanomaterials interacting with metal nanomaterials constitute abundant heterostructures, which can modulate and enhance the light‐matter interaction of materials via active plasmonic effects. These modulation results in heterostructures can be concluded into two parts depending on optical signals. On the one hand, metallic nanomaterials can enhance or quench the PL intensity of 2D semiconductors. Spectra splitting of shift can also be observed in strong coupling process of exciton and surface plasmon. On the other hand, the heterostructures employed as sensors can significantly enhance the fingerprint Raman spectra of molecules and other biological samples. Due to extraordinary properties of 2D nanomaterials and the flexibility of parameters control as reviewed above, the potential applications are of growing interest in many fields as highly compacted light emitting devices, sensitive optical sensors, and efficient chemical catalyst driven by light power.

### 4.1 PL Modulation

**Figure 7:**
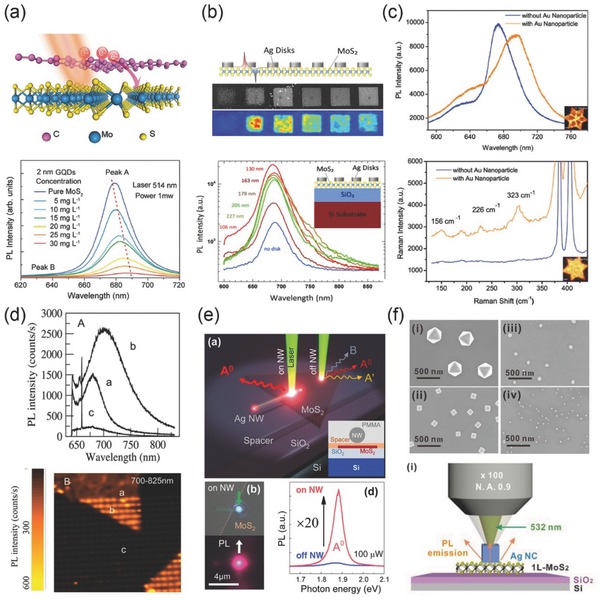
a) Upper: graphene quantum dots and monolayer MoS_2_ heterostructure and the charge transfer illustration, bottom: PL spectrum of MoS_2_ changes with the GQDs concentration. Reproduced with permission.^[113]^ b) Upper: sketch, SEM image and PL mapping of Ag‐MoS_2_ hybrid structure, bottom: PL spectrum intensity is influenced by Ag disks modification as well as the diameter of nanodisk. (Inset) cross‐section view of the sample design. Reproduced with permission.^[117]^ Copyright 2015, ACS. c) Upper: PL spectrum of 5nm diameter Au NPs deposited MoS_2_ and pristine 2D MoS_2_ sheet, lower: characteristic Raman spectrum of Au‐MoS_2_ hybrid structure. Reproduced with permission.^[118]^ d) Upper: PL spectra of MoS_2_ on SiO_2_/Si substrate, MoS_2_ on gold nanoantenna and bare gold nanoantenna, lower: corresponding PL intensity mapping. Reproduced with permission.^[119]^ Copyright 2014, ACS. e): Upper: schematics of launching and propagation of SPPs coupled with excitons, bottom left: optical image of the hybrid structure illuminated by laser, bottom right: PL spectral of MoS_2_ samples with and without nanowires. (Inset): cross‐section of structure design. Reproduced with permission.^[121]^ Copyright 2015, APS. f) Upper: SEM images of four types of Ag NPs, lower: schematics of Ag NPs modified PL measuring method. Reproduced with permission.^[123]^

The optical properties of metal/2D materials heterostructures benefit greatly from the localized strong electric field induced by surface plasmon. The electric field contributes to the strength of light‐matter interaction resulting in absorption enhancement.^109^, ^110^, ^111^, ^112^ Excited electrons of 2D semiconductors are generated from light absorption, then relax to the bottom of conductance band, bind into exciton energy level, and finally decay in relaxation process which includes non‐radiative and radiative decay. In addition, the plasmonic effect can modulate the decay process, which is important for light‐emitting and thermal lattice vibration. Hence, the interface problem of heterostructures is quite important and sensitive to optical properties of 2D materials. Charge transfer and energy transfer occur at heterostructure interface and can further result in PL spectra tuning, involving intensity attenuation and signal enhancement. It is demonstrated that an effective charge transfer process is recorded in atomically thin GQD/MoS_2_ heterostructure observing from the spectral evolution of MoS_2_ PL with increasing GQDs concentration (**Figure** **7**a).^113^ PL spectra decreases and redshifts step by step when GQDs solution is spin‐coated onto MoS_2_ monolayer again and again. From a three‐energy‐level model, the doped carrier density (6.5 × 10^13^ cm^−2^) can be estimated from the optical intensity, which changes with the doping concentration. This tuning effect is further used to control the degree of MoS_2_ valley polarization at different GQDs deposition densities. The total PL intensity can be decomposed into exciton and trion recombination. Exciton emission efficiency is sensitive with doping charges, but trion is insensitive with doping charges, because the spectral evolution arises from the competition of exciton and trion recombination. Compared with electric gate doping and chemical doping, it is proved to be an efficient n‐type doping by fabricating 0D/2D heterostructures. These results pave the way for the exploration of light–matter interactions in 2D nanomaterials.

Charge transfer is directly affected by the spacer thickness between the metallic nanomaterials and 2D semiconductors. Generally, the decay process may even happen in negligible spacing with few nanometer thickness. When an appropriate spacer layer is precisely designed to separate the direct contact of metal and materials, charge transfer may be blocked, and the non‐radiative transition will be restrained. A manipulation method of the PL intensity from nearly 4‐fold quenching to approximately 3‐fold enhancement is achieved by adding a spacer layer between MoSe_2_ layer and nanoantennas.^114^ Plasmonic dipolar antennas are excited in resonant condition at 532 nm. The exciton‐plasmon interaction is manipulated by varying nanorods length ranging from 70 to 130 nm in a square lattice. Furthermore, the coupled system exhibits strong polarization‐dependent selective PL enhancement, which provides the possibility in active control of polarization‐based emission. This work offers an important way to understand the important role of spacer layer for large‐range PL manipulation from quenching to enhancement based on 2D semiconductors.

Another example of restraining the non‐radiative decay is reported by A. Sobhani and his colleagues.^115^ In the structure frame of inserting dielectric layer, silica–Au–PVP (poly‐4‐vinylpyridine) core–shell NPs were utilized to separate metal and semiconductor. PVP layer acts as an insulating layer to block the non‐radiative process efficiently. The main absorption enhancement is obviously observed at 645 nm benefiting greatly from the strong plasmonic resonance. Light‐induced oscillations of free electrons in metallic nanostructure strengthen the light‐matter interaction. As a result, a nearly twice times enhancement of PL intensity was obtained. To further increase the PL emission, a hybrid structure consisting of Ag nanocube/PVP/MoS_2_/HfO_2_/Au film was reported by G. M. Akselrod, which shows remarkable PL enhancement of WSe_2_ up to 2000‐fold.^116^ The nanocube with dielectric layer has two main resonance, one is the fundamental dipole mode at 660 nm and the other is the second‐order mode at 420 nm. The giant PL enhancement is realized by leveraging two separated resonant modes, which provides a flexible and tunable plasmonic platform to control the optical process of 2D materials.

With the rapid development of CVD growth technology, centimeter‐sized MoS_2_ flakes with high‐quality are fabricated. Large‐area fabrication of nanoparticles allowed us to investigate the interaction between the exciton PL and the plasmonic resonance of the particles. Large–size PL enhancement of CVD MoS_2_ is reported when it couples with plasmonic nanodisk arrays of which the diameters range from 106 to 227 nm (Figure 7b).^117^ Results show that enhanced PL intensity of MoS_2_ film directly depends on localized E‐field intensity generated by LSPR. The maximum enhancement of PL signal is achieved as 12‐fold, which arises from both the excitation field enhancement at the pump wavelength and the efficient optical scattering of nandisks. Efficient light harvesting and light emission of these low‐dimensional materials with large‐size area can open up new ground in integrated optoelectronic devices with considerable performance.

Besides the PL intensity changing, peak broadening and energy shifting are also observed due to the plasmonic effect using different contact interfaces. Some researchers reported that plasmonic hot electrons can induce phase transition of MoS_2_ monolayer at 77 K low temperature (Figure 7c).^118^ 5 nm Au NPs were spin‐coated and deposited onto MoS_2_ film forming metal‐semiconductor heterostructure. Hot electrons are generated by plasmonic resonant excitation with a high energy at 2.56 eV, which is much higher than the Schottky barrier (0.8 eV) between MoS_2_ and Au NPs. 2H‐MoS_2_ shows semiconductor properties with high stability at room temperature, which is due to the field‐induced splitting of Mo *4d*‐orbitals of a D_3h_‐MoS_6_ unit into three groups. Hot electrons can jump through the barrier, dope the MoS_2_ monolayer, lead to a lattice destabilization, through the population of Mo 4d‐orbitals, and then into 1T phase. 1T‐MoS_2_ possesses incomplete occupation of degenerate orbitals, which shows metallic properties. In addition, plasmonic effects also result in spectral broadening and red‐shifting as shown in the figure. The main reason of this tuning is that if hot electrons transfer into MoS_2_ semiconductor within a non‐radiative decay, the band gap of MoS_2_ is narrowed. The method of off‐ and in‐resonance control offers selective enhancement of MoS_2_ PL emission.^119^ In a hybrid structure of Au nanoantenna arrays and MoS_2_ monolayer as shown in Figure 7d, the relation of PL intensity amplification with localized temperature distribution has been investigated, which is caused by the plasmonic mediated light absorption. The PL spectra also show broadening and shifting during plasmonic induced heat process. In the experiment, the temperature of MoS_2_ above the nanoantenna array is measured by a temperature calibration procedure reaching up to 120 °C ±20 °C. It is nearly two times larger than the temperature of MoS_2_ without antenna contact. In theoretical prediction, the thermal effects can be interpreted in terms of plasmonic induced light absorption and its conversion into electron‐hole pair in MoS_2_, which is verified by using green dyadic method (GDM) and Discrete Dipole Approximation (DDA) simulations combined with heat dissipation calculations.

Surface plasmon polaritons (SPPs) are collective oscillations of free electrons in metal, which can propagate along the interface of metal and dielectric. Plasmonic waveguides propagating SPPs can realize integrated photonic circuits showing high‐frequency operation rate. Excitons in quantum dots coupling with SPPs are widely reported in the last ten years, such as single‐photon SPPs and quantum coherent. Recent research averts their sights on 2D semiconductor exciton coupling with SPPs. J. Kim's group reported a series of work on exciton‐plasmon conversion in Ag nanowire/MoS_2_ (Figure 7e). MoS_2_ PL is of multi‐channel exciton, involving primary exciton, charge exciton and valence splitting induced exciton. The primary exciton plays dominant role in total contribution of PL. H. S. Lee demonstrated a clever method realizing selectively amplifying the primary exciton of MoS_2_ monolayer in a cavity‐free hybrid system based on cyclic re‐excitation exciton.^120^, ^121^ The nanowire length effect was systematically investigated by controlling fully and partially overlapping of MoS_2_ film. For fully overlapped samples, the PL enhancement is independent with the laser incident position. However, the laser position is significant in the situation of partially overlapped region. The maximum of enhancement factor reaches nearly 20 selectively for the primary exciton, which excludes other multi‐exciton by significantly reduced band filling effect.

The mechanism is that exciton couples directly with the input laser, and the enhanced primary exciton emission recouples SPPs times and times. This work opens up a shortcut to realize potential TMD optoelectronics coupling with SPPs, and provides a new model of selective PL amplification in recycle principle. Besides the PL enhancement, the SPPs propagation can also be real‐space visualized benefits from the MoS_2_ gain medium. The atomically flat and thin properties provide fewer scattering losses of SPPs propagation in Ag nanowire.

The PL enhancement is strongly decided on field enhancement, collection efficiency and quantum efficiency.^122^ The large field enhancement excited at resonant wavelength acts as high‐index to increase optical absorption, promotes electron‐hole pairs generation and accelerates the radiative rate. The overall collection efficiency is related with objective lens and the overlapped area of nanoparticles and 2D semiconductors. Quantum efficiency depends on the position and dipole moment of exciton in the electric field. Tailoring all the three components in hybrid system provides a platform to realize the PL gaining control at the nanoscale. A heterostructure is demonstrated consisting of shape‐controlled Ag nanoantenna and mechanically exfoliated MoS_2_ film.^123^ The PL intensity of 1L‐MoS_2_ can be continuously tuned from huge enhancement to slight attenuation by changing the morphologies of Ag nanoantenna as shown in Figure 7f. Ag nanoantennas, involving Ag octahedron (Ag OTC), sphere (Ag SP) and nanocube (Ag NC) show various diameters range from 40 nm to 340 nm, and their main resonant peaks observed from extinction spectra change from 400 to 1000 nm with different sizes. In the situation of Ag NC‐MoS_2_, the localized resonance of nanoantenna overlaps the excitation energy and MoS_2_ band gap, which results in dramatic PL enhancement up to 950‐fold. The quantum yield calculated by electrodynamic theory is estimated to 13.9‐fold at the strongest enhancement situation, and the photon collection efficiency slightly decreases from 15% to 13% due to the cover of Ag particles. However, PL weakening is observed in Ag OCT‐MoS_2_ structure, where the non‐radiative high‐order plasmon plays the dominant role. The field enhancement is still large and calculated as 280‐fold, but the quantum yield and collection efficiency is reduced to 0.2‐fold and 0.26%, respectively. Considering all the effects together, the final PL enhancement is decreased to 0.89‐fold.

### 4.2 Photodetection

2D materials are appealing candidates for a variety of optoelectronic devices because of its ultrathin thickness and distinctive characteristics, which are suitable for transparent electrodes, photovoltaic and photodetection. Among those potential applications, great efforts have been devoted to photodetectors based on monolayer graphene and MoS_2_. Graphene is a gapless material, which enables a large‐range light absorption over ultraviolet, visible, infrared and terahertz spectral regimes. The linear dispersion property of graphene provides tunable optical absorption according to n‐type and p‐type doping conditions. The ultrafast carrier mobility and low dissipation rates enable ultrafast conversion and ultrasensitive detection of photons. Besides graphene, LTMDs are a big family with unique electronic and optoelectronic properties. LTMDs are direct band gap semiconductors, which are suitable for light harvesting without momentum dissipation. Because of their distinctive properties as transparency, mechanical flexibility and tunable band gap, LTMDs can offer particular advantages in strong light absorption in visible range. A promising development trend is the combination of plasmonic nanomaterials and 2D crystal heterogeneous stacks, which benefits plasmonic effects and ultrathin thickness resulting in strong light‐matter interaction and sensitive photon detection.

The physical mechanism of photodetection based on 2D semiconductors is mainly among the photovoltaic effect, photo‐thermoelectric effect and bolometric effect. The generation of photovoltaic current is arising from the separation of light‐induced electron‐hole pairs by built‐in electric field or source‐drain The built‐in electric field is associated with localized doping and heterojunction with different work‐function. The direction of built‐in electric field determines the photocurrent direction, and it is independent with the overall doping level. The photo‐thermoelectric effect can be generated by hot carriers, which plays a dominant role in graphene p‐n junction or suspended graphene. Because photoexcited e‐h pairs will be transformed into hot electrons due to ultrafast process and strong e‐e scattering. The bolometric effect is based on light‐induced inhomogeneous thermal distribution, which is arising from the change of carrier mobility with variant temperature. In addition, other physical mechanisms can also create photocurrent, including the photogating effect and the plasma‐wave‐assisted mechanism.^124^, ^125^, ^126^ They each can be happened in a practical photodetector, and it is worthy to be understood when each of them may become dominant.

**Figure 8:**
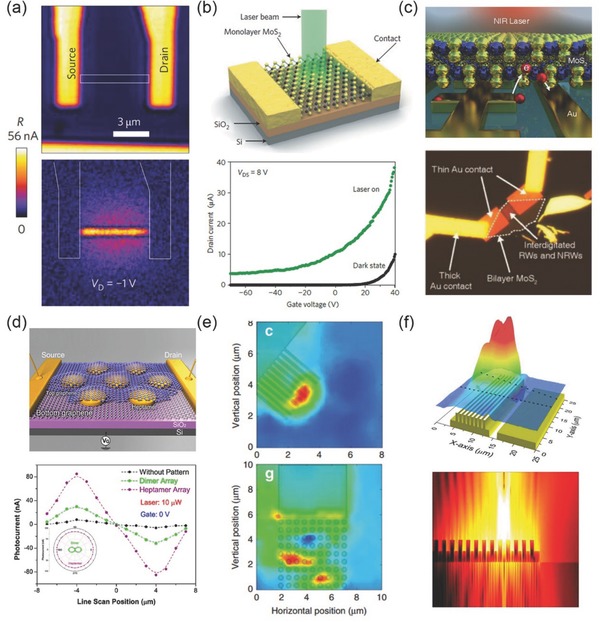
a) Illustration of graphene photodetector with gate bias. Reproduced with permission.^[127]^ Copyright 2012, Nature Publishing Group. b) Upper: schematic view of monolayer MoS_2_ based photodetector, bottom: gating response in dark/illuminated state. Reproduced with permission.^[129]^ Copyright 2013, Nature Publishing Group. c) Plasmonic structures coupled bilayer MoS_2_ photodetection. Reproduced with permission.^[130]^ Copyright 2015, ACS. d) Upper: illustration of nanoantennas sandwiched graphene photodetector, bottom: PL spectral as the function of line scan position. (Inset) polarization dependent photocurrent response. Reproduced with permission.^[131]^ Copyright 2012, ACS. e) Two kinds of plasmonic nanostructure enhanced graphene photodetectors. f) Surface plasmon polaritons coupled graphene photodetector. (e,f) Reproduced with permission.^[134]^ Copyright 2015, ACS.

The mechanism of intrinsic photoresponse of homogeneous graphene with gate bias tuning was reported (**Figure** **8**a).^127^ The origin of photoresponse of grapheme has been studied in details. A homogeneous graphene ribbon was fabricated on Si/SiO_2_ substrate acting as the channel of field effect transistor. In this classic photodetective device, the difference of photocurrent polarities due to photovoltaic and thermoelectric effects are investigated, which are opposite and allow us to directly judge the dominant role with gate bias changing. First, they investigated the thermoelectric effect in a p‐n junction at zero bias. Next, the bolometric response can be determined by measuring the temperature‐dependent current in a gate‐biased device. Finally, a photovoltaic and photo‐induced bolometric effect dominates the photoresponse, which is as large as ten‐times in magnitude compared with the thermoelectric effect. The characteristic photocurrent can be modulated by electrostatic doping of graphene, which offers a proper way to probe the hot‐carriers induced photoresponse and heat dissipation of phonons. Another way to break the mirror symmetry of built‐in potential in graphene device is inducing an asymmetric metallization. The first graphene photodetector is demonstrated in a 10 Gbit s^−1^ optical data link.^128^ The maximum external photoresponsivity was achieved as 6.1 mA W^−1^ at the wavelength of 1.55 um.

TMDs with direct band gap can offer additional advantages in photodetection at visible spectral range due to quantum‐mechanical confinement. Benefiting from their layer and mechanical properties, flexible optoelectronic devices of TMDs show great potential in photodetection. With the development of TMDs grown technology, a variety of 2D semiconductor photodetectors have been widely reported performing distinctive superiority as shown in Figure 8b. Photodetectors based on other 2D materials show respective photoresponse, such as GaTe (10^4^ A W^−1^), GaSe (2.8 A W^−1^), GaS (19 A W^−1^), In_2_Se_3_ (3.95 A W^−1^), WS_2_ (22 uA W^−1^). Besides, the first monolayer MoS_2_ photodetector exhibits a low photoresponsivity of 7.5 mA W^−1^, which is at the same level of graphene devices (6.1 mA W^−1^). The multilayer MoS_2_ photodetector shows a higher photoresponse up to 100 mA W^−1^, which can compete with Silicon‐based devices. In 2003, an ultrasensitive monolayer MoS_2_ photodetector with a photoresponsivity as high as 880 A W^−1^ at 561 nm was demonstrated, which shows 100,000‐fold enhancement than previous reported.^129^ At a low laser power of 0.15 uW, the dark current increased from 0 to 4 uA. The ratio of photocurrent intensity at on‐ and off‐ state was observed more than 4 when the bias volatage was controlled at 8 V. The photoresponsivity is ultrasensitive spanning from 400 nm to 680 nm due to the band gap absorption. This exciting results arise from the improvement of electron mobility in MoS_2_ monolayer, as well as the contact quality and positioning technique.

The intrinsic absorption of 2D materials is determined by their electronic band gap, which results in photoresponse in a limited spectral range. The heterostructures of metal/2D semiconductor can expand the optical absorption in a large spectral range due to plasmonic effects. The mechanism of surface plasmon enhanced photodetection can be understood in two ways. On the one hand, plasmonic resonances of metal particles are able to generate a large amount of hot electrons, jump and tunnel the potential barrier, and inject into 2D semiconductors resulting in photocurrent. On the other hand, the near‐field enhancement induced by plasmonic effect will enhance the photon absorption contributing to enhanced light‐matter interaction (Figure 8c). A near‐infrared MoS_2_ photodetector shows unique interest which is comprised of plasmonic metallic nanostructures.^130^ The plasmonic nanostructure consists of resonant and non‐resonant wires, where the dipolar resonant absorption occurs at 1250 nm. To avoid indirect photon absorption of bilayer MoS_2_ at 750 nm (1.65 eV), the incident laser energy is chosen at a much lower energy. Therefore, the photocurrent comes from the hot electrons generated by resonant wire coupling. The mechanism of hot electron induced photocurrent amplification was verified by changing the bias polarity. The relative contribution of hot electrons injection and photothermoelectric effect can be distinguished by the Schottky barrier. The large photogain, as large as 10^5^‐fold enhancement, results in a photoresponsivity of 5.2 A W^−1^ at 1070 nm, which is comparable with similar silicon‐based hot electron optoelectronic device.

Plasmonic Fano resonance has been investigated for a few decades, which is the spectral interference between a broad and narrow resonance. Because the resonant field enhancement is extraordinary high, and the narrow Fano line‐shape is sensitive with the environment, it is quite attractive for 2D photodetection with plasmonic Fano resonant effect. An efficient graphene photodetector was designed by sandwiching plasmonic heptamer antennas between two single layer graphene films (Figure 8d).^131^ The heptamer antennas consist of seven nanodisks with the diameter varying from 80 nm to 180 nm, corresponding to a Fano resonant frequency from 650 to 950 nm. The photocurrent of antenna sandwitched graphene photodetector is observed as high as 80 nA, which is 800‐fold larger than the antennaless graphene device. And the internal quantum efficiency is achieved up to 20% in the visible and near‐infrared spectral range. Besides, there are many similar hybrid devices based on plasmonic nanostructures. Some researchers fabricated graphene photodetector coupled with nanodisks, dimers and gratings by using e‐beam lithography.^132^ The photovoltage enhancement can be increased by up to 20 times the plasmonic induced field concentration at the junction. In addition, the polarization dependent photoresponse can be selectively enhanced by employing nanostructures of different geometries. A new strategy to realize plasmon resonance enhanced multicolor photodetection is also demonstrated.^133^ A back gated graphene transistors are first fabricated on SiO_2_/Si substrate, then the thermal annealed nanoparticles was transferred by PMMA film. With the random plasmonic nanoparticles, the photocurrent amplitude is increased up to 2.2 mA W^−1^ with an average enhancement of the photoresponsivity by more than 400%. This is because of the subwavelength light scattering and plasmonic resonant absorption effect. It shows that plasmonic enhanced graphene photodetectors can greatly enhance the photocurrent and external quantum efficiency by up to 1,500%. And the selective photodetection, among 500 to 650 nm can be realized by varying gold nanoparticles size, enabling highly specific detection of multicolor.

Other types of photodetection devices based on 2D materials and plasmonics have been reported (Figure 8e,f). A plasmon‐assisted *h‐*BN/Graphene/WS_2_/Graphene stacked 2D photodetectors shows an enhanced external quantum efficiency as high as 30% due to the strong light‐matter interaction in these atomically thin layers.^134^ The two graphene layers acting as contact electrode, and can be individually adjusted via gate bias tuning. The plasmonic nanospheres acting as optical resonators are further utilized to increase photoresponse, where the optical field in the active layer are dramatically enhanced allowing for a 10‐fold increase in the photocurrent. They also reported surface plasmon polaritons coupled graphene photodetectors combing plasmonic gratings with graphene transistors. Laser excitation can be achieved by fabricating metallic diffraction gratings, and SPPs deliver the energy to the contact region of a metal‐graphene‐metal, which further result in the overall absorption. Based on the coupling mechanism, a 400% enhancement of responsivity and a 1000% increase are achieved with tunable spectral selectivity below 50 nm bandwidth. A graphene photodetection method based on ionic liquid gated plasmonic Ag nanoparticle is reported, which takes advantages of surface plasmon enhanced light harvest and ionic liquid induced high doping efficiency.^135^ The photoreponsivity is obtained as large as 350 mA W^−1^ at the resonant wavelength of LSPR.

### 4.3 Photocatalysis

Semiconductors are the most studied photocatalytic materials which are promising to achieve mass applications of photocatalysis. However, no single semiconductor material can realize this goal due to the intrinsic shortcomings.^136^, ^137^ Take TiO_2_ as an example, it has many advantages such as earth‐abundant and nontoxicity, while it suffers from a severe weakness—too wide bandgap (3.2 eV). This means the onset wavelength that TiO_2_ can absorb is about 390 nm. TiO_2_ can only make use of ultraviolet light which represents 5% of solar spectrum energy. Therefore, the efficiency of TiO_2_ is undoubtedly low in photocatalysis. In contrast, one of the reasons why single layer MoS_2_ have aroused great attention is that it is a direct bandgap semiconductor and its bandgap is suitable (1–2 eV).^20^, ^138^ To overcome the defects of semiconductors, researchers have introduced other materials like dyes into photocatalysis. A recent new promising approach is integrating plasmonic materials with semiconductors and the most common structure in plasmon‐enhanced photocatalysis is metal‐semiconductor heterojunction.^19^, ^139^, ^140^

There are mainly three mechanisms involved in the field of plasmon‐enhanced photocatalysis. The first one relies on plasmonic induced hot electrons.^141^, ^142^ After the excitation of localized surface plasmon resonances (LSPR), the free electrons oscillate collectively at the metal surface. As a consequence of Landau damping, the oscillation in phase gradually becomes diphase and then forms a non‐equilibrium electron distribution. Consequently, electron‐electron scattering can exchange the energy between high‐energy electrons and those with low energy, leading to a Fermi‐Dirac‐like electron distribution—hot electron distribution. This process is completed in timescale of a few hundred femtoseconds. Next, the hot electrons produced by the decay of surface plasmons interact with the lattice or phonons, transferring electron energy to phonons. Therefore, to efficiently take advantage of hot electrons in catalyzing chemical reactions like water splitting, it is of prime importance to export plasmonic hot electrons before its decaying. A part of hot electrons generated by the dephasing of surface plasmons are able to overcome the Schottky barrier. These electrons increase the occupation of semiconductor's conduction band and will move to the interface of semiconductor/electrolyte solution, participating in catalytic reactions. The second mechanism is associated with the enhanced near field caused by surface plasmon resonances. The electric field at the surface of metal nanostructures can be largely enhanced when LSPR is excited. The field intensity decreases exponentially with the distance away from metal surface. When the semiconductor is close to metal nanoparticles, the electric field intensity inside the semiconductor is dramatically increased, resulting in enhanced light absorption. Thus the number of generated electron‐hole pairs in semiconductors is significantly raised. Herein, 2D semiconducting materials show their potential for deeper analysis and more effective modulations of plasmonic properties because they have single atomic layer thickness in one dimension. Besides, their shapes are easier to be adjusted, which allows for fully touch in metal‐semiconductor heterojunction like coating metal nanoparticles with 2D semiconductors. However, one request must be satisfied to utilize this mechanism that the plasmon band is required to be overlapping with semiconductor's bandgap. Hence, the near field mechanism can only work when the energy of LSPR band is higher than bandgap energy. The third mechanism is based on far‐field effect or scattering effect. The scattering cross section rises as the size of metal nanoparticle increases. From the perspective of geometrical optics, the light not absorbed by semiconductor can be scattered by metal nanoparticles consecutively. Therefore, the light path in semiconductor is multiply increased, causing enhanced absorption in semiconductor. Similarly, this mechanism only makes effects when the energy of scattering light exceeds bandgap energy.

As the demand for energy is increasing, it is necessary to substitute renewable and sustainable resources for fossil fuels. Photocatalysis which converts solar energy into chemical energy has attracted great interest. Semiconductor metal oxides are the most often used photocatalytic materials, although the efficiency is far from the level for practical application. Recently, researchers pay great attention to 2D materials for their utilization in photocatalysis due to novel properties, such as high charge mobility rate. Surface plasmons, which can enhance the absorption of visible light, are integrated to improve the performance of 2D materials.

**Figure 9:**
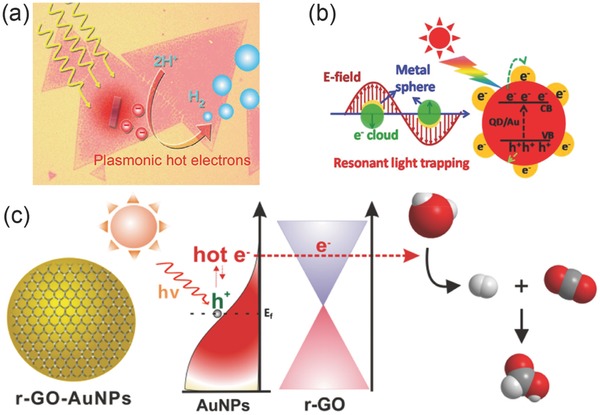
a) Schematic view of single layer MoS_2_ catalysis in hydrogen evolution reaction supported by plasmonic hot electron. Reproduced with permission.^[20]^ Copyright 2015, RSC. b) The illustration of localized SPR effect induced charge separation and transfer process in heteronanocrystals supported by graphene. Reproduced with permission.^[19]^ Copyright 2014, ACS. c) Graphene‐coated gold nanoparticles and the schematics showing photoconversion mechanism of CO_2_ into HCOOH. Reproduced with permission.^[143]^ Copyright 2016, ACS.

The introduction of plasmonic nanorattles effectively enhances the performance of molybdenum disulfide (MoS_2_) monolayer in hydrogen evolution reaction (HER). Some researchers deposited Ag nanorattles coated with Au on MoS_2_ monolayer (shown as **Figure** **9**a), and they found the photocatalytic efficiency was improved by virtue of plasmonic hot electron doping.^20^ The core‐shell nanostructure was chosen because of its narrow absorbance linewidth. Besides, as Ag is very likely to oxidize in water environment, the Au shell could prevent it from oxidization. When illuminated by 690 nm laser, corresponding to the wavelength of surface plasmon resonances, the HER activity was increased. From the polarization curves of HER, the current density was dramatically increased, rising from ≈75 uA*cm^−2^ (excited by off‐resonance 532 nm laser) to ≈125 uA*cm^−2^ (excited by on‐resonance 690 nm laser). Furthermore, the authors plotted Tafel curve and obtained that the slope declined from ≈175 mV per decade to ≈155 mV per decade, which illustrated that the reaction rate was accelerated by exciting surface plasmon resonances. Based on this result, they believed that plasmonic hot electron played an important role in the improved performance. They supposed that the doping of plasmonic hot electrons and the stress induced by nanorattles caused the structure phase change of MoS_2_, resulting in better photocatalytic activity. According to their former work, 2H phase MoS_2_ would transform into 1T phase with the doping of plasmonic hot electrons. In this work, the new raised Raman peaks of MoS_2_ deposited with nanorettles confirmed that the 1T phase was produced. Furthermore, they studied the influence of laser power in HER performance. With the increasing of laser power, they found the onset potential gradually decreased and current density went up. This result was accounted that more plasmonic hot electrons were generated when illuminated by higher power incident light. In addition, they derived the phase transition irreversible from the fact that the improved activity was sustained when the laser power is decreased.

The degradation of organic dyes is of critical significance in environmental protection. Some researchers reported a photocatalyst that could not only enhance the activity of H_2_ generation from water splitting, but also improve the performance in organic dye degradation.^19^ They first synthesized CdSe/CdS nanocrystals and then deposited Au nanoparticles (NPs) on them, forming CdSe/CdS‐Au core‐satellite heterostructure. Next, they assembled the heterocrystals on graphene nanosheet, which could further enhance the photocatalytic activity. The CdSe/CdS core‐shell quantum dots (QDs) were chosen due to the good photostability. In this case, LSPR of Au NPs was excited by visible light and the enhanced near field promoted the formation of electron‐hole pairs in QDs. The schematic diagram was shown in Figure 9b. At the surface of QDs, electrons were excited to the conduction band and transferred to Au satellites, while holes remained at the QDs, which generated different photocurrent responses depending on whether having hole scavenger. On the condition that there was free of hole scavenger in electrolyte solution (NaNO_3_), the generated holes moved to the electrode and produced cathode current. While there exit hole scavenger (Na_2_S and Na_2_SO_3_) in solution, the holes would be depleted and the electrons transferred to the anodic electrode, resulting in adverse photocurrent response. By comparing the absorption spectra of the heteronanocrystals with control groups comprised of discrete QDs and Au NPs, they found that the spectra were not simply the sum of these parts and deduced that there was interaction between surface plasmons and excitons.

Next, they studied the photodegradation performance of QD/Au‐G for methylene blue (MB) and Rhodamine B (RDB). For both kinds of dyes, QD/Au‐G showed better ability in photodegradation than the reference groups. From this result, it was not difficult to infer the active effect of plasmon. As for H_2_ production from water splitting, QD/Au‐G composite also demonstrated best activity among all tested photocatalyst. Besides, the H_2_ evolution rate was dramatically improved with the help of graphene, which illustrated the positive role of graphene in accelerating H_2_ generation. In this work, graphene promoted the efficiency because of three causes. The first is graphene has good water‐solubility. Secondly, graphene has excellent conductivity. Therefore, the electrons in QDs could be efficiently transferred, leading to decreased recombination rate. Thirdly, graphene provided more active absorption and reaction sites. However, increasing the content of graphene could not continuously enhance photocatalytic activity as too much graphene could hinder the absorption of incident light and lessened the active reaction sites. The most suitable graphene content was about 1%. The maximum H_2_ generation rate was 3113 µmol h^‐1^ g^‐1^ and the quantum yield was estimated to be 25.4% at 450 nm. This work mainly took advantage of the enhanced near field produced by LSPR, which improved the absorption of QDs and thus facilitated the generation of electron‐hole pairs.

Another important photocatalytic reaction is the reduction of CO_2_, which realizes the depletion of greenhouse gas CO_2_ and generation of useful products like methane (CH_4_) and formic acid (HCOOH). A high‐efficiency CO_2_ conversion photocatalyst that consisted of graphene coated Au nanoparticles (r‐GO‐AuNPs) was synthetized as shown in Figure 9c.^143^ The photoconversion was proposed to take place by two steps. Firstly, the hot electrons induced by the irradiative decay of localized surface plasmons transferred to water molecule. H_2_ molecules were then generated by water splitting. Secondly, the produced H_2_ reacted with CO_2_, forming HCOOH and methanol (CH_3_OH). The UV‐visible spectra showed that Pt‐Au NPs and r‐GO‐AuNPs had almost same plasmon peak, locating at 540 nm. In addition, the extinction spectra did not change after illuminated by Xe lamp for three hours, and r‐GO‐AuNPs showed a litter better performance than Pt‐AuNPs in catalytic efficiency, indicating that r‐GO was possible to substitute for Pt. Meanwhile, this catalyst illustrated excellent production selectivity with HCOOH content higher than 90%. From the experiments measuring the quantum and chemical yield of different photocatalyst, r‐GO‐AuNPs demonstrated best activity with its efficiency far exceeding r‐GO (7.6 times) under the irradiation of Xe lamp. However, the quantum and chemical field of r‐GO‐AuNPs were not dramatically increased when excited by NIR (808 nm) laser, because surface plasmons were not resonantly excited. These experimental results disclosed that both plasmonic material and graphene played positive roles in raising quantum efficiency. The reaction time was estimated to be about 3 hours and the optimum pH was 9.0 for the photoconversion. Interestingly, increasing r‐GO‐AuNPs concentration adequately resulted in better yield, while too much content led to declined efficiency. To mimic natural solar illumination, they measured the quantum and chemical yields under AM 1.5 filter. The result showed much lower yields due to the power density passed solar filter (0.15 W/cm^2^) was less than that of Xe lamp (5.68 W/cm^2^).

To fully explore the contribution of Au NPs, they carried out femtosecond transient absorption experiments on r‐GO‐AuNPs/TiO_2_ composite. The pump pulse wavelength was 575 nm and the wavenumber of probe light was 2978 cm^−1^. They discovered that the plasmonic hot electron transfer process was completed within 220 fs as the transient absorption signal reached maximum at that time after excitation. Similar to the results of quantum and chemical yield, the peak signals of r‐GO‐AuNPs and GO‐AuNPs were 8.7 and 2.8 times higher than the value of pure AuNPs, respectively. In this work, graphene acted as electron acceptor and transporter because the plasmon‐induced hot electrons were firstly moved to the coated graphene layer before transferring to water molecules.

## 5 Conclusion and Outlook

In this review, we summarized the plasmonics of 2D nanomaterials, which include 2D materials as graphene, LTMDCs and hybridization with nanoparticles as well as nanostructures. Based on their unique properties and coupling effects, we proposed several research directions for the light‐matter interaction studies, plasmonic tuning methods and potential applications in the future.

First, we elaborated the advanced electronic structures and optical properties of 2D materials. The plasmonic properties of 2D materials were further introduced by analyzing the oscillating system of Dirac Fermions. Based on latest studies, we discussed the coupling effect achieved by the LTMDCs/metal nanostructure hybrid system, and the multi‐oscillators model which is promising for analyzing complicated system of more resonant modes. Moreover, controlling configuration properties such as size, contour, position, and crystalline structures is the key point to the practical applications. Thus, a new generation of efficient and accurate fabrication technique is very in need, and new methods for active material manipulating are also required to realize more functional modulations.^144^, ^145^ On the one hand, latest configuration method is reported to reduce oxygen functional groups from graphene oxide into pristine graphene, by the simple and efficient method of microwave pulses instead of high‐cost CVD technique, 2D materials are closer to practical applications.^146^ On the other hand, a new CVD process without introducing seed layers is inspiring, which enables the high‐quality production of monolayer 2D materials.^147^ However, the extraordinary properties of 2D materials are still not ideal and stable in the experimental environment, more profound studies are in dire need. A recent nanomechanical testing result reveals the fragile behavior of large‐area freestanding MoSe_2_ membrane, which may come from inherent defects of 2D materials induced by the configuration or transfer procedure.^148^ Thus, more proofs should be made for the applications of flexible electronics because the experimental elastic strain of semiconducting LTMDCs and metallic graphene may not be as high as we expected. Interestingly, a Reactive Force Field Method (ReaxFF) is recently utilized to analyze and control the defects induced properties of graphene by simulating interactions of quantities of atoms forced by external perturbations.^149^ Since the collective oscillations of electrons are also correlated with crystal defects, this method can be important for practical plasmonic utilizations of functional 2D nanomaterials.

Then, we reviewed recent researches for the plasmonic tuning of 2D nanomaterials. The types of doping are summarized including electron injection, gas physisorption, and chemical methods, which can tune plasmonic properties by changing the carrier density. According to the boundary condition of SPP, the plasmonic mode of 2D materials can be modulated by constructing it into specific pattern. Furthermore, the hybridization of 2D materials and metal nanomaterials attracts growing interest, since the metallic plasmons interact strongly with excitons and 2D material plasmons. 2D materials hybrid structures such as ZnO/graphene or LTMDCs/photonic crystal compound stacks show potential to be photodiode for photocurrent generation or photodetection,^150^ furthermore, latest SPs induced nanolasers methods attract great interest, and many groups are searching for a way to create ultra‐small, tunable, thresholdless and efficient laser sources which can be promising for high‐resolution medical imaging and on‐chip optical communications.^151^, ^152^ However, due to the different atomic arrangements and bonding types between metal and 2D materials, the interfacial condition are hard to be controlled. More details about interfacial processes including charge transfer, energy transfer and coupling interaction need to be further explored. Moreover, unique properties of 2D nanomaterials compound stacks cannot be well explained by classical charge transport theory for bulk semiconductors, the advanced characteristics of ultrathin 2D nanomaterials compound stacks should be studied more profoundly. In particular, a systematic study of layer‐dependent studies is in need because of the complexity and highly variable nature of the van der Waals interfaces between different 2D materials layers and uncontrollable extrinsic factors in configuration procedures.^153^

Finally, we elaborated plasmon‐induced applications of 2D nanomaterials. Owing to the ultra‐thin nature and strong light‐emitting properties, 2D nanomaterials have great potential for polarized LEDs and optical labels. 2D materials hybridized with metal nanostructures are also promising for optoelectronic devices such as transparent electrodes, photovoltaic devices and photodetectors. The photocatalytic reactions driven by light are based on the excitation of hot carriers between the interface of metal and semiconductor, which are believed to be promising for environmental friendly energy generation in the future. With the continuing researches on printed electronics based on 2D nanomaterials, inspiring applications are found including wearable sensors and flexible transistors, recently, a method of UV‐pulsed laser technique is carried out to selectively irradiate reduced graphene oxide (RGO) while keeping the high electrical conductivity in the paper‐based circuit. By combining the 3D printing technology, it is even possible to develop graphene/3D nanostructures beyond this planar circuit to enable more favorable possibilities.^154^ However, the device parameters for 2D nanomaterials in the nanoscale region are hard to be unified in large manufacturing process resulting from the complicated configuration steps, the way to practical industrial production is still long. For applications of photocatalysis, the charge transfer procedure is always under great concern. The ultra‐fast charge transfer phenomena in LTMDCs/plasmonic metasurface hybrid structures measured by pump–probe spectroscopy are recently reported, which can inspire further studies as ultrafast optical switching of graphene SPs.^138^, ^155^ But most reports about plasmon‐induced hot electron catalytic reactions are about metal and traditional semiconductors rather than 2D nanomaterials, quantified explanations of the ultra‐fast transfer procedure in the hybrid system are still waiting to be discovered.

## References

[1] K. F. Mak , C. Lee , J. Hone , J. Shan , T. F. Heinz , Phys. Rev. Lett. 2010, 105, 136805. 21230799 10.1103/PhysRevLett.105.136805

[2] S. Dai , Z. Fei , Q. Ma , A. S. Rodin , M. Wagner , A. S. McLeod , M. K. Liu , W. Gannett , W. Regan , K. Watanabe , Science 2014, 343, 1125. 24604197 10.1126/science.1246833

[3] Y. Lin , T. V. Williams , J. W. Connell , J. Phys. Chem. Lett. 2009, 1, 277.

[4] B. Radisavljevic , A. Radenovic , J. Brivio , V. Giacometti , A. Kis , Nat. Nanotechnol. 2011, 6, 147. 21278752 10.1038/nnano.2010.279

[5] A. K. Geim , K. S. Novoselov , Nat. Mater. 2007, 6, 183. 17330084 10.1038/nmat1849

[6] K. S. Novoselov , A. K. Geim , S. V. Morozov , D. Jiang , Y. Zhang , S. V. Dubonos , I. V. Grigorieva , A. A. Firsov , Science 2004, 306, 666. 15499015 10.1126/science.1102896

[7] F. Bonaccorso , Z. Sun , T. Hasan , A. C. Ferrari , Nat. Photonics 2010, 4, 611.

[8] R. R. Nair , P. Blake , A. N. Grigorenko , K. S. Novoselov , T. J. Booth , T. Stauber , N. M. R. Peres , A. K. Geim , Science 2008, 320, 1308. 18388259 10.1126/science.1156965

[9] M. D. Stoller , S. Park , Y. Zhu , J. An , R. S. Ruoff , Nano Lett. 2008, 8, 3498. 18788793 10.1021/nl802558y

[10] Y. Zhang , Y. W. Tan , H. L. Stormer , P. Kim , Nature 2005, 438, 1. 10.1038/nature04235 16281031

[11] K. F. Mak , L. Ju , F. Wang , T. F. Heinz , Solid State Commun. 2012, 152, 1341.

[12] T. Eberlein , U. Bangert , R. R. Nair , R. Jones , M. Gass , A. L. Bleloch , K. S. Novoselov , A. Geim , P. R. Briddon , Phys. Rev. B 2008, 77, 233406.

[13] Z. Fei , G. O. Andreev , W. Bao , L. M. Zhang , S. M. A , C. Wang , M. K. Stewart , Z. Zhao , G. Dominguez , M. Thiemens , M. M. Fogler , M. J. Tauber , A. H. Castro‐Neto , C. N. Lau , F. Keilmann , D. N. Basov , Nano Lett. 2011, 11, 4701. 21972938 10.1021/nl202362d

[14] Y. Liu , R. F. Willis , K. V. Emtsev , T. Seyller , Phys. Rev. B 2008, 78.

[15] K. S. Novoselov , D. Jiang , F. Schedin , T. J. Booth , V. V. Khotkevich , S. V. Morozov , A. K. Geim , Proc. Natl. Acad. Sci. USA 2005, 102, 10451. 16027370 10.1073/pnas.0502848102 PMC1180777

[16] Q. Bao , K. P. Loh , ACS Nano 2012, 6, 3677. 22512399 10.1021/nn300989g

[17] A. Molina‐Sánchez , L. Wirtz , Phys. Rev. B 2011, 84, 155413.

[18] Q. H. Wang , K. Kalantarzadeh , A. Kis , J. N. Coleman , M. S. Strano , Nat. Nanotechnol. 2012, 7, 699. 23132225 10.1038/nnano.2012.193

[19] J. Zhang , P. Wang , J. Sun , Y. Jin , ACS Appl. Mater. Interfaces 2014, 6, 19905. 25369420 10.1021/am505371g

[20] Y. Kang , Y. Gong , Z. Hu , Z. Li , Z. Qiu , X. Zhu , P. M. Ajayan , Z. Fang , Nanoscale 2015, 7, 4482. 25682885 10.1039/c4nr07303g

[21] S. Zu , Y. Bao , Z. Fang , Nanoscale 2016, 8, 3900. 26818746 10.1039/c5nr09302c

[22] H. Zhu , F. Yi , E. Cubukcu , Nat. Photonics 2016, 10, 709.

[23] A. J. Haes , D. A. Stuart , S. Nie , R. P. Van Duyne , J. Fluoresc. 2004, 14, 355. 15617378 10.1023/b:jofl.0000031817.35049.1f

[24] D. G. Baranov , S. V. Makarov , A. E. Krasnok , P. A. Belov , A. Alù , Laser Photonics Rev. 2016, 10, 1009.

[25] Y. Yu , Z. Ji , S. Zu , B. Du , Y. Kang , Z. Li , Z. Zhou , K. Shi , Z. Fang , Adv. Funct. Mater. 2016, 26, 6394.

[26] S. F. Shi , T. T. Tang , B. Zeng , L. Ju , Q. Zhou , A. Zettl , F. Wang , Nano Lett. 2014, 14, 1578. 24564302 10.1021/nl404826r

[27] S. Wang , S. Li , T. Chervy , A. Shalabney , S. Azzini , E. Orgiu , J. A. Hutchison , C. Genet , P. Samorì , T. W. Ebbesen , Nano Lett. 2016, 16, 14368. 10.1021/acs.nanolett.6b01475 27266674

[28] H. Y. Jeong , U. J. Kim , H. Kim , G. H. Han , H. Lee , M. S. Kim , Y. Jin , T. H. Ly , S. Y. Lee , Y. G. Roh , W. J. Joo , S. W. Hwang , Y. Park , Y. H. Lee , ACS Nano 2016, 10, 8192. 27556640 10.1021/acsnano.6b03237

[29] A. H. C. Neto , Phys. Rev. Lett. 2000, 86, 4382.

[30] Q. Bao , H. Zhang , Y. Wang , Z. Ni , Y. Yan , Z. X. Shen , K. P. Loh , D. Y. Tang , Adv. Funct. Mater. 2009, 19, 3077.

[31] Z. Sun , T. Hasan , F. Torrisi , D. Popa , G. Privitera , F. Wang , F. Bonaccorso , D. M. Basko , A. C. Ferrari , ACS Nano 2009, 4, 803. 10.1021/nn901703e 20099874

[32] T. Kampfrath , L. Perfetti , F. Schapper , C. Frischkorn , M. Wolf , Phys. Rev. Lett. 2005, 95, 187403. 16383946 10.1103/PhysRevLett.95.187403

[33] A. Kuc , N. Zibouche , T. Heine , Phys. Rev. B 2011, 83, 245213.

[34] C. Lee , H. Yan , L. E. Brus , T. F. Heinz , J. Hone , S. Ryu , ACS Nano 2010, 4, 2695. 20392077 10.1021/nn1003937

[35] B. L. Evans , P. A. Young , P. Roy. Soc. A‐Math. Phy. 1965, 284, 402.

[36] D. Y. Qiu , F. H. da Jornada , S. G. Louie , Phys. Rev. Lett. 2013, 111, 216805. 24313514 10.1103/PhysRevLett.111.216805

[37] Y. Lin , X. Ling , L. Yu , S. Huang , A. L. Hsu , Y. H. Lee , J. Kong , M. S. Dresselhaus , T. Palacios , Nano Lett. 2014, 14, 5569. 25216267 10.1021/nl501988y

[38] H. Shi , R. Yan , S. Bertolazzi , J. Brivio , B. Gao , A. Kis , D. Jena , H. G. Xing , L. Huang , ACS Nano 2013, 7, 1072. 23273148 10.1021/nn303973r

[39] H. Wang , C. Zhang , W. Chan , C. Manolatou , S. Tiwari , F. Rana , Phys. Rev. B 2016, 93, 045407.

[40] D. Xiao , G. B. Liu , W. Feng , X. Xu , W. Yao , Phys. Rev. Lett. 2012, 108, 196802. 23003071 10.1103/PhysRevLett.108.196802

[41] G. Eda , H. Yamaguchi , D. Voiry , T. Fujita , M. Chen , M. Chhowalla , Nano Lett. 2011, 11, 5111. 22035145 10.1021/nl201874w

[42] T. Okada , A. Okamura , Y. Kosuge , IEEE T. Thz. Sci. Techn. 2013, 3, 63.

[43] R. S. Sundaram , M. Engel , A. Lombardo , R. Krupke , A. C. Ferrari , P. Avouris , M. Steiner , Nano Lett . 2013, 13, 1416. 23514373 10.1021/nl400516a

[44] G. Moody , J. Schaibley , X. Xu , J. Opt. Soc. Am. B 2016, 33, C39. 10.1364/JOSAB.33.000C39 PMC5590662 28890600

[45] S. Mouri , Y. Miyauchi , K. Matsuda , Nano Lett. 2013, 13, 5944. 24215567 10.1021/nl403036h

[46] S. Boubanga‐Tombet , S. Chan , T. Watanabe , A. Satou , V. Ryzhii , T. Otsuji , Phys. Rev. B 2012, 85, 035443.

[47] H. Karasawa , T. Komori , T. Watanabe , A. Satou , H. Fukidome , M. Suemitsu , V. Ryzhii , T. Otsuji , J. Infrared, Millim. Te. 2010, 32, 655.

[48] W. L. Barnes , A. Dereux , T. W. Ebbesen , Nature 2003, 424, 824. 12917696 10.1038/nature01937

[49] T. W. Ebbesen , H. J. Lezec , H. F. Ghaemi , T. Thio , P. A. Wolff , Nature 1998, 391, 667.

[50] Z. Fang , X. Zhu , Adv Mater 2013, 25, 3840. 23813594 10.1002/adma.201301203

[51] F. J. García de Abajo , Rev. Mod. Phys. 2007, 79, 1267.

[52] A. N. Grigorenko , M. Polini , K. S. Novoselov , Nat. Photonics 2012, 6, 749.

[53] P. Tassin , T. Koschny , M. Kafesaki , C. M. Soukoulis , Nat. Photonics 2012, 6, 259.

[54] L. Brey , H. A. Fertig , Phys. Rev. B 2007, 75, 125434.

[55] S. D. Sarma , E. H. Hwang , Phys. Rev. Lett. 2009, 102, 206412. 19519055 10.1103/PhysRevLett.102.206412

[56] O. L. Berman , R. Y. Kezerashvili , K. G. Ziegler , Physics 2011, 85, 276.

[57] R. R. Hartmann , J. Kono , M. E. Portnoi , Nanotechnology 2014, 25, 3. 10.1088/0957-4484/25/32/322001 25051014

[58] S. Gangadharaiah , A. M. Farid , E. G. Mishchenko , Phys. Rev. Lett. 2008, 100, 166802. 18518232 10.1103/PhysRevLett.100.166802

[59] K. F. Mak , M. Y. Sfeir , Y. Wu , C. H. Lui , J. A. Misewich , T. F. Heinz , Phys. Rev. Lett. 2008, 101, 196405. 19113291 10.1103/PhysRevLett.101.196405

[60] J. C. Idrobo , W. Zhou , J. Lee , J. Nanda , S. T. Pantelides , S. J. Pennycook , Nat. Nanotechnol. 2012, 7, 161. 22286496 10.1038/nnano.2011.252

[61] Z. Q. Li , E. A. Henriksen , Z. Jiang , Z. Hao , M. C. Martin , P. Kim , H. L. Stormer , D. N. Basov , Nat. Phys. 2008, 4, 532.

[62] P. Vasa , W. Wang , R. Pomraenke , M. Lammers , M. Maiuri , C. Manzoni , G. Cerullo , C. Lienau , Nat. Photonics 2013, 7, 128.

[63] A. Bostwick , F. Speck , T. Seyller , K. Horn , M. Polini , R. Asgari , A. H. Macdonald , E. Rotenberg , Science 2010, 328, 999. 20489018 10.1126/science.1186489

[64] H. Walther , B. Varcoe , B. Englert , T. Becker , Rep. Prog. Phys. 2006, 69, 377.

[65] P. Törmä , W. L. Barnes , Rep. Prog. Phys. 2015, 78, 013901. 25536670 10.1088/0034-4885/78/1/013901

[66] N. A. Mortensen , S. Xiao , Appl. Phys. Lett. 2007, 90, 141108.

[67] M. D. Lukin , A. Imamoglu , Phys. Rev. Lett. 1999, 84, 137. 10.1103/PhysRevLett.84.1419 11017532

[68] S. Wang , A. Mika , J. A. Hutchison , C. Genet , A. Jouaiti , M. W. Hosseini , T. W. Ebbesen , Nanoscale 2014, 6, 7243. 24898976 10.1039/c4nr01971g

[69] J. Kasprzak , M. Richard , S. Kundermann , A. Baas , P. Jeambrun , J. M. Keeling , F. M. Marchetti , M. H. Szymańska , R. André , J. L. Staehli , Nature 2006, 443, 409. 17006506 10.1038/nature05131

[70] E. Orgiu , J. George , J. A. Hutchison , E. Devaux , J. F. Dayen , B. Doudin , F. Stellacci , C. Genet , J. Schachenmayer , C. Genes , Nat. Mater. 2015, 14, 219. 10.1038/nmat4392 26366850

[71] X. Liu , T. Galfsky , Z. Sun , F. Xia , E. C. Lin , Y. H. Lee , S. Kéna‐Cohen , V. M. Menon , Nat. Photonics 2014, 9, 30.

[72] M. Brune , F. Schmidt‐Kaler , A. Maali , J. Dreyer , E. Hagley , J. M. Raimond , S. Haroche , Phys. Rev. Lett. 1996, 76, 1800. 10060524 10.1103/PhysRevLett.76.1800

[73] N. T. Fofang , T. H. Park , O. Neumann , N. A. Mirin , P. Nordlander , N. J. Halas , Nano Lett. 2008, 8, 3481. 18729410 10.1021/nl8024278

[74] A. I. Väkeväinen , R. J. Moerland , H. T. Rekola , A. P. Eskelinen , J. P. Martikainen , D. H. Kim , P. Törmä , Nano Lett. 2014, 14, 1721. 24279840 10.1021/nl4035219

[75] J. Dintinger , S. Klein , F. Bustos , W. L. Barnes , T. W. Ebbesen , Phys. Rev. B 2005, 71, 035424.

[76] S. R. Rodriguez , J. Feist , M. A. Verschuuren , F. J. Garcia Vidal , R. J. Gómez , Phys. Rev. Lett. 2013, 111, 166802. 24182291 10.1103/PhysRevLett.111.166802

[77] L. Shi , T. K. Hakala , H. T. Rekola , J. P. Martikainen , R. J. Moerland , P. Törmä , Phys. Rev. Lett. 2014, 112, 153002. 24785036 10.1103/PhysRevLett.112.153002

[78] V. G. Kravets , F. Schedin , A. N. Grigorenko , Phys. Rev. Lett. 2008, 101, 2444. 10.1103/PhysRevLett.101.087403 18764660

[79] W. Liu , B. Lee , C. H. Naylor , H. S. Ee , J. Park , A. T. Johnson , R. Agarwal , Nano Lett. 2016, 16, 1262. 26784532 10.1021/acs.nanolett.5b04588

[80] N. K. Emani , A. V. Kildishev , V. M. Shalaev , A. Boltasseva , Nanophotonics 2015, 4, 214.

[81] F. H. Koppens , D. E. Chang , F. J. Garcia de Abajo , Nano Lett. 2011, 11, 3370. 21766812 10.1021/nl201771h

[82] Y. Wang , J. Z. Ou , A. F. Chrimes , B. J. Carey , T. Daeneke , M. M. Alsaif , M. Mortazavi , S. Zhuiykov , N. Medhekar , M. Bhaskaran , J. R. Friend , M. S. Strano , K. Kalantar‐Zadeh , Nano Lett. 2015, 15, 883. 25562610 10.1021/nl503563g

[83] V. W. Brar , M. S. Jang , M. Sherrott , S. Kim , J. J. Lopez , L. B. Kim , M. Choi , H. Atwater , Nano Lett. 2014, 14, 3876. 24874205 10.1021/nl501096s

[84] B. Radisavljevic , A. Kis , Nat. Mater. 2013, 12, 815. 23793161 10.1038/nmat3687

[85] E. J. Lee , K. Balasubramanian , R. T. Weitz , M. Burghard , K. Kern , Nat. Nanotechnol. 2008, 3, 486. 18685636 10.1038/nnano.2008.172

[86] J. Chen , M. Badioli , P. Alonso‐Gonzalez , S. Thongrattanasiri , F. Huth , J. Osmond , M. Spasenovic , A. Centeno , A. Pesquera , P. Godignon , A. Z. Elorza , N. Camara , F. J. Garcia de Abajo , R. Hillenbrand , F. H. Koppens , Nature 2012, 487, 77. 22722861 10.1038/nature11254

[87] Z. Fei , A. S. Rodin , G. O. Andreev , W. Bao , A. S. McLeod , T. M. Slipchenko , M. Wagner , L. M. Z , Z. Zhao , M. Thiemens , G. Dominguez , M. M. Fogler , A. H. Castro Neto , C. N. Lau , F. Keilmann , D. N. Basov , Nature 2012, 487, 82. 22722866 10.1038/nature11253

[88] Z. Fang , S. Thongrattanasiri , A. Schlather , Z. Liu , L. Ma , Y. Wang , P. M. Ajayan , P. Nordlander , N. J. Halas , G. D. A. Fj , ACS Nano 2013, 7, 2388. 23390960 10.1021/nn3055835

[89] Z. Fang , Y. Wang , A. E. Schlather , Z. Liu , P. M. Ajayan , F. J. de Abajo , P. Nordlander , X. Zhu , N. J. Halas , Nano Lett. 2014, 14, 299. 24320874 10.1021/nl404042h

[90] A. Y. Nikitin , P. Alonsogonzález , S. Vélez , S. Mastel , A. Centeno , A. Pesquera , A. Zurutuza , F. Casanova , L. E. Hueso , F. H. L. Koppens , Nat. Photonics 2016, 10, 239.

[91] L. Ju , B. Geng , J. Horng , C. Girit , M. Martin , Z. Hao , H. A. Bechtel , X. Liang , A. Zettl , Y. R. Shen , Nat. Nanotechnol. 2011, 6, 630. 21892164 10.1038/nnano.2011.146

[92] J. Christensen , A. Manjavacas , S. Thongrattanasiri , F. H. Koppens , F. J. de Abajo , ACS Nano 2011, 6, 431. 22147667 10.1021/nn2037626

[93] N. K. Emani , D. Wang , T.‐F. Chung , L. J. Prokopeva , A. V. Kildishev , V. M. Shalaev , Y. P. Chen , A. Boltasseva , Laser Photonics Rev. 2015, 9, 650.

[94] D. Ansell , I. P. Radko , Z. Han , F. J. Rodriguez , S. I. Bozhevolnyi , A. N. Grigorenko , Nat. Commun. 2015, 6, 8846. 26554944 10.1038/ncomms9846 PMC5227092

[95] W. Cao , V. Pankratov , M. Huttula , X. Shi , S. Saukko , Z. Huang , M. Zhang , Mater. Chem. Phys. 2015, 158, 89.

[96] S. Zu , B. Li , Y. Gong , Z. Li , P. M. Ajayan , Z. Fang , Adv. Opt. Mater. 2016, 4, 1463.

[97] C. H. Liu , I. S. Kim , L. J. Lauhon , Nano Lett. 2015, 15, 6727. 26356284 10.1021/acs.nanolett.5b02586

[98] J. Mertens , A. L. Eiden , D. O. Sigle , F. Huang , A. Lombardo , Z. Sun , R. S. Sundaram , A. Colli , C. Tserkezis , J. Aizpurua , S. Milana , A. C. Ferrari , J. J. Baumberg , Nano Lett. 2013, 13, 5033. 24059599 10.1021/nl4018463

[99] N. Dabidian , I. Kholmanov , A. B. Khanikaev , K. Tatar , S. Trendafilov , S. H. Mousavi , C. Magnuson , R. S. Ruoff , G. Shvets , ACS Photonics 2015, 2, 216.

[100] N. K. Emani , T. F. Chung , A. V. Kildishev , V. M. Shalaev , Y. P. Chen , A. Boltasseva , Nano Lett. 2014, 14, 78. 24303876 10.1021/nl403253c

[101] B. Zhao , Z. M. Zhang , ACS Photonics 2015, 2, 1611.

[102] Y. Bao , S. Zu , Y. Zhang , Z. Fang , ACS Photonics 2015, 2, 1135.

[103] J. Kim , H. Son , D. J. Cho , B. Geng , W. Regan , S. Shi , K. Kim , A. Zettl , Y. R. Shen , F. Wang , Nano Lett 2012, 12, 5598. 23025816 10.1021/nl302656d

[104] N. K. Emani , T. F. Chung , X. Ni , A. V. Kildishev , Y. P. Chen , A. Boltasseva , Nano Lett 2012, 12, 5202. 22950873 10.1021/nl302322t

[105] B. D. Thackray , P. A. Thomas , G. H. Auton , F. J. Rodriguez , O. P. Marshall , V. G. Kravets , A. N. Grigorenko , Nano Lett. 2015, 15, 3519. 25859743 10.1021/acs.nanolett.5b00930

[106] G. Xu , J. Liu , Q. Wang , R. Hui , Z. Chen , V. A. Maroni , J. Wu , Adv. Mater. 2012, 24, OP71. 22392746 10.1002/adma.201104846

[107] M. Engel , M. Steiner , A. Lombardo , A. C. Ferrari , H. V. Lohneysen , P. Avouris , R. Krupke , Nat . Commun. 2012, 3, 906. 22713748 10.1038/ncomms1911 PMC3621428

[108] S. Heeg , R. Fernandez‐Garcia , A. Oikonomou , F. Schedin , R. Narula , S. A. Maier , A. Vijayaraghavan , S. Reich , Nano Lett. 2013, 13, 301. 23215014 10.1021/nl3041542

[109] C. Janisch , H. Song , C. Zhou , Z. Lin , A. L. Elías , D. Ji , M. Terrones , Q. Gan , Z. Liu , 2D Mater. 2016, 3, 025017.

[110] J. Lee , S. Shim , B. Kim , H. S. Shin , Chemistry 2011, 17, 2381. 21264961 10.1002/chem.201002027

[111] K. C. Lee , Y. H. Chen , H. Y. Lin , C. C. Cheng , P. Y. Chen , T. Y. Wu , M. H. Shih , K. H. Wei , L. J. Li , C. W. Chang , Sci. Rep. 2015, 5, 16374. 26576041 10.1038/srep16374 PMC4647184

[112] X. Li , W. C. H. Choy , X. Ren , D. Zhang , H. Lu , Adv. Funct. Mater. 2014, 24, 3114.

[113] Z. Li , R. Ye , R. Feng , Y. Kang , X. Zhu , J. M. Tour , Z. Fang , Adv. Mater. 2015, 27, 5235. 26255655 10.1002/adma.201501888

[114] H. Chen , J. Yang , E. Rusak , J. Straubel , R. Guo , Y. W. Myint , J. Pei , M. Decker , I. Staude , C. Rockstuhl , Y. Lu , Y. S. Kivshar , D. Neshev , Sci. Rep. 2016, 6, 22296. 26923211 10.1038/srep22296 PMC4770425

[115] A. Sobhani , A. Lauchner , S. Najmaei , C. Ayala‐Orozco , F. Wen , J. Lou , N. J. Halas , Appl. Phys. Lett. 2014, 104, 031112.

[116] G. M. Akselrod , T. Ming , C. Argyropoulos , T. B. Hoang , Y. Lin , X. Ling , D. R. Smith , J. Kong , M. H. Mikkelsen , Nano Lett. 2015, 15, 3578. 25914964 10.1021/acs.nanolett.5b01062

[117] S. Butun , S. Tongay , K. Aydin , Nano Lett. 2015, 15, 2700. 25729895 10.1021/acs.nanolett.5b00407

[118] Y. Kang , S. Najmaei , Z. Liu , Y. Bao , Y. Wang , X. Zhu , N. J. Halas , P. Nordlander , P. M. Ajayan , J. Lou , Z. Fang , Adv. Mater. 2014, 26, 6467. 25100132 10.1002/adma.201401802

[119] S. Najmaei , A. Mlayah , A. Arbouet , C. Girard , J. Léotin , J. Lou , ACS Nano 2014, 8, 12682. 25469686 10.1021/nn5056942

[120] H. S. Lee , M. S. Kim , Y. Jin , G. H. Han , Y. H. Lee , J. Kim , Adv. Opt. Mater. 2015, 3, 943.

[121] H. S. Lee , M. S. Kim , Y. Jin , G. H. Han , Y. H. Lee , J. Kim , Phys Rev Lett 2015, 115, 226801. 26650314 10.1103/PhysRevLett.115.226801

[122] S. Y. Choi , C. T. Yip , G.‐C. Li , D. Y. Lei , K. H. Fung , S. F. Yu , J. Hao , AIP Adv. 2015, 5, 067148.

[123] W. Gao , Y. H. Lee , R. Jiang , J. Wang , T. Liu , X. Y. Ling , Adv. Mater. 2016, 28, 701. 26607311 10.1002/adma.201503905

[124] T. Hong , B. Chamlagain , S. Hu , S. M. Weiss , Z. Zhou , Y. Q. Xu , ACS Nano 2015, 9, 5357. 25871507 10.1021/acsnano.5b01065

[125] G. Eda , S. A. Maier , ACS Nano 2013, 7, 5660. 23834654 10.1021/nn403159y

[126] T. Mueller , F. Xia , P. Avouris , Nat. Photonics 2010, 4, 297.

[127] M. Freitag , T. Low , F. Xia , P. Avouris , Nat. Photonics 2012, 7, 53.

[128] F. H. Koppens , T. Mueller , P. Avouris , A. C. Ferrari , M. S. Vitiello , M. Polini , Nat. Nanotechnol. 2014, 9, 780. 25286273 10.1038/nnano.2014.215

[129] O. Lopezsanchez , D. Lembke , M. Kayci , A. Radenovic , A. Kis , Nat. Nanotechnol. 2013, 8, 497. 23748194 10.1038/nnano.2013.100

[130] W. Wang , A. Klots , D. Prasai , Y. Yang , K. I. Bolotin , J. Valentine , Nano Lett. 2015, 15, 7440. 26426510 10.1021/acs.nanolett.5b02866

[131] Z. Fang , Z. Liu , Y. Wang , P. M. Ajayan , P. Nordlander , N. J. Halas , Nano Lett. 2012, 12, 3808. 22703522 10.1021/nl301774e

[132] T. J. Echtermeyer , L. Britnell , P. K. Jasnos , A. Lombardo , R. V. Gorbachev , A. N. Grigorenko , A. K. Geim , A. C. Ferrari , K. S. Novoselov , Nat. Commun. 2011, 2, 458. 21878912 10.1038/ncomms1464

[133] U. J. Kim , S. Yoo , Y. Park , M. Shin , J. Kim , H. Jeong , C.‐W. Baik , Y.‐G. Roh , J. Lee , K. Im , H. Son , S. Hwang , C.‐W. Lee , S. Park , ACS Photonics 2015, 2, 506.

[134] T. Echtermeyer , S. Milana , U. Sassi , A. Eiden , M. Wu , E. Lidorikis , A. C. Ferrari , Nano Lett. 2015, 16, 8. 26666842 10.1021/acs.nanolett.5b02051

[135] G. Xu , R. Lu , J. Liu , H.‐Y. Chiu , R. Hui , J. Z. Wu , Adv. Opt. Mater. 2014, 2, 729.

[136] B. Luo , G. Liu , L. Wang , Nanoscale 2016, 8, 6904. 26961514 10.1039/c6nr00546b

[137] S. Mubeen , J. Lee , N. Singh , S. Krämer , G. D. Stucky , M. Moskovits , Nat. Nanotechnol. 2013, 8, 247. 23435280 10.1038/nnano.2013.18

[138] Y. Yu , Z. Ji , S. Zu , B. Du , Y. Kang , Z. Li , Z. Zhou , K. Shi , Z. Fang , Adv. Funct. Mater. 2016, 26, 6394.

[139] A. Hoggard , L. Y. Wang , L. Ma , Y. Fang , G. You , J. Olson , Z. Liu , W. S. Chang , P. M. Ajayan , S. Link , ACS Nano 2013, 7, 11209. 24266755 10.1021/nn404985h PMC3932108

[140] H. Zhang , A. O. Govorov , J. Phys. Chem. C. 2014, 118, 7606.

[141] Z.‐g. Dai , X.‐h. Xiao , W. Wu , Y.‐p. Zhang , L. Liao , S.‐s. Guo , J.‐j. Ying , C.‐x. Shan , M.‐t. Sun , C.‐z. Jiang , Light‐Sci. Appl. 2015, 4, e342.

[142] N. M. Gabor , P. Jarillo‐Herrero , Science 2011, 334, 648. 21979935 10.1126/science.1211384

[143] D. Kumar , A. Lee , T. Lee , M. Lim , D. K. Lim , Nano. Lett. 2016, 16, 1760. 26854830 10.1021/acs.nanolett.5b04764

[144] J. Annett , G. L. Cross , Nature 2016, 535, 271. 27411633 10.1038/nature18304

[145] P. Mulpur , S. Yadavilli , A. M. Rao , V. Kamisetti , R. Podila , ACS Sensors 2016, 1, 826.

[146] D. Voiry , J. Yang , J. Kupferberg , R. Fullon , C. Lee , H. Y. Jeong , H. S. Shin , M. Chhowalla , Science 2016, 353, 1414. 10.1126/science.aah3398 27708034

[147] A. Alharbi , D. Shahrjerdi , Appl. Phys. Lett. 2016, 109, 193502.

[148] Y. Yang , X. Li , M. Wen , E. Hacopian , W. Chen , Y. Gong , J. Zhang , B. Li , W. Zhou , P. M. Ajayan , Q. Chen , T. Zhu , J. Lou , Adv. Mater. 2016, 29, 1604201. 10.1002/adma.201604201 27809368

[149] K. Yoon , A. Rahnamoun , J. L. Swett , V. Iberi , D. A. Cullen , I. V. Vlassiouk , A. Belianinov , S. Jesse , X. Sang , O. S. Ovchinnikova , A. J. Rondinone , R. R. Unocic , A. C. van Duin , ACS Nano 2016, 10, 8376. 27532882 10.1021/acsnano.6b03036

[150] H. Zhu , X. Xu , X. Tian , J. Tang , H. Liang , L. Chen , Y. Xie , X. Zhang , C. Xiao , R. Li , Q. Gu , P. Hua , S. Ruan , Adv. Mater. 2016, 29, 1604351. 10.1002/adma.201604351 27862431

[151] C. Palacios‐Berraquero , M. Barbone , D. M. Kara , X. Chen , I. Goykhman , D. Yoon , A. K. Ott , J. Beitner , K. Watanabe , T. Taniguchi , A. C. Ferrari , M. Atature , Nat. Commun. 2016, 7, 12978. 27667022 10.1038/ncomms12978 PMC5052681

[152] S. Wu , S. Buckley , J. R. Schaibley , L. Feng , J. Yan , D. G. Mandrus , F. Hatami , W. Yao , J. Vuckovic , A. Majumdar , X. Xu , Nature 2015, 520, 69. 25778703 10.1038/nature14290

[153] W. J. Yu , Q. A. Vu , H. Oh , H. G. Nam , H. Zhou , S. Cha , J. Y. Kim , A. Carvalho , M. Jeong , H. Choi , A. H. Castro Neto , Y. H. Lee , X. Duan , Nat. Commun. 2016, 7, 13278. 27827360 10.1038/ncomms13278 PMC5105192

[154] S. R. Das , Q. Nian , A. A. Cargill , J. A. Hondred , S. Ding , M. Saei , G. J. Cheng , J. C. Claussen , Nanoscale 2016, 8, 15870. 27510913 10.1039/c6nr04310k

[155] G. X. Ni , L. Wang , M. D. Goldflam , M. Wagner , Z. Fei , A. S. McLeod , M. K. Liu , F. Keilmann , B. Özyilmaz , A. H. C. Neto , Nat. Photonics 2016, 10, 244.

